# Gland cell responses to feeding in *Drosera capensis*, a carnivorous plant

**DOI:** 10.1007/s00709-021-01667-5

**Published:** 2021-06-21

**Authors:** Irene Lichtscheidl, Sue Lancelle, Marieluise Weidinger, Wolfram Adlassnig, Marianne Koller-Peroutka, Sonja Bauer, Stefanie Krammer, Peter K. Hepler

**Affiliations:** 1 Cell Imaging and Ultrastructure Research, University of Vienna, Althanstrasse 14, A-1090 Vienna, Austria; 2 Biology Department, University of Massachusetts Amherst, 221 Morrill Science Center III; 611 North Pleasant Street, Amherst, MA 01003-9297 USA

**Keywords:** Gland cell, *Drosera capensis*, Carnivorous

## Abstract

Glands of *Drosera* absorb and transport nutrients from captured prey, but the mechanism and dynamics remain unclear. In this study, we offered animal proteins in the form of fluorescent albumin (FITC-BSA) and observed the reactions of the glands by live cell imaging and fluorescence microscopy. The ultrastructure of these highly dynamic processes was also assessed in high-pressure frozen and freeze substituted (HPF-FS) cells. HPF-FS yielded excellent preservation of the cytoplasm of all cell types, although the cytosol looked different in gland cells as compared to endodermoid and stalk cells. Especially prominent were the ER and its contacts with the plasma membrane, plasmodesmata, and other organelles as well as continuities between organelles. Also distinct were actin microfilaments in association with ER and organelles. Application of FITC-BSA to glands caused the formation of fluorescent endosomes that pinched off the plasma membrane. Endosomes fused to larger aggregates, and accumulated in the bulk cytoplasm around the nucleus. They did not fuse with the cell sap vacuole but remained for at least three days; in addition, fluorescent vesicles also proceeded through endodermoid and transfer cells to the epidermal and parenchymal cells of the tentacle stalk.

**Supplementary Information:**

The online version contains supplementary material available at 10.1007/s00709-021-01667-5.

## Introduction

*Drosera* is one of the largest and most diverse genera of carnivorous plants (CPs). The structure and physiology of these plants have been of great interest ever since Darwin (1875) described their carnivorous nature, not only because of the exceptional way they acquire nutrition, but also because of their economic importance in pharmacy due to their antimicrobial metabolites and their sticky, trapping glue (Grevenstuk et al. 2009; Sprague-Piercy et al. 2020). Plants are characterized by leaves bearing protuberances on the margin and upper surface of their blades; they consist of a slender stalk, or tentacle, that bears a multicellular glandular head. In addition, several types of small glandular trichomes occur on the leaf blade and the tentacle stalk (Naidoo and Heneidak 2013). The glands of the tentacles secrete a sticky mucilage that lures insects and holds them tight. The tentacles further enwrap the prey, which is degraded by secreted digestive enzymes. The tentacles then absorb the digested nutrients, as they actively move over the prey. This remarkable multiple function of the *Drosera* tentacles has made them an important object of investigations about general questions of morphology, physiology, ecology, and evolution of carnivorous plants. Despite a wealth of information, which has been summarized (Lloyd 1942; Juniper et al. 1989; Ellison and Adamec 2018), many vexing questions about absorption and transport of prey substances remain unsolved.

Although questions have been raised concerning the benefit of prey consumption (Daubenmire 1974), even early studies suggested that *Drosera* profits from carnivorous nutrition by the formation of additional flowers (Francis Darwin 1878), and more recently that those plants have a faster rate of growth (Chandler and Anderson 1976a, b). Studies have further shown that minerals are absorbed from digested prey (Adamec 1997; Shibata and Komiya 1972, 1973) and that the plant nutrient status and photosynthetic performance were increased (Pavlovic et al*.* 2014; Pavlovic and Saganova 2015).

Concerning secretion and absorption in *Drosera* gland cells, the Golgi apparatus has been shown to play a major role in the production and secretion of the trapping mucilage (Schnepf 1961, 1969; Outenreath and Dauwalder 1986). The production and secretion of lytic enzymes associated with feeding have also received considerable attention (see Dexheimer 1976, 1978; Outenreath and Dauwalder 1982, 1986; McNally et al. 1988; Muravnik 2000; Plachno et al. 2006). Further studies on the absorption of fluorescently labeled proteins showed that nutrients can enter the cells by endocytosis, in addition to transport through the plasma membrane (Adlassnig et al. 2012). Membrane recycling to pre-vacuolar compartments and vacuoles occurs for arabinogalactan proteins as detected by immunolocalization (Samaj et al. 2000).

For further transport of nutrients from glands to leaves, different routes through the tentacle stalk have been suggested as being either through the central xylem strand or through the inner or the epidermal stalk cells (Juniper et al*.* 1989); however, definitive experiments are still missing. Nevertheless, the conclusion that stalk cells absorb nutrients is strongly supported by the observation of “aggregation,” a remarkable reaction first described by Darwin (1875), which includes swelling of the cytoplasm and acceleration of organelle movement.

The ultrastructure study of gland cells has yielded important information about Golgi bodies and the production of mucilage (Schnepf 1961; Ragetli et al. 1972; Dexheimer 1972, 1976, 1978; Gilchrist and Juniper 1974; Juniper and Gilchrist 1976; Outenreath and Dauwalder 1982, 1986; Muravnik 2000); however, they fall short in representing faithfully the instantaneous situation of organelle interactions and membrane organization during various physiological activities. Especially the cells of the tentacle stalk, which are covered by a thick cuticle, have been extremely difficult to preserve due to long fixation times; fixation can take more than ten minutes (our own observations) and lead to structural reorganizations and reorientation of the cytoplasm and its organelles, a phenomenon that becomes even more important in deeper layers of the tissue.

In this study, we investigated the ultrastructure of *Drosera capensis* glands during absorption and transport of proteins, a highly dynamic process as seen in studies using fluorescently labeled proteins (Adlassnig et al. 2012), and applied freeze fixation techniques for faithful reflection of the in vivo state in unstimulated glands and in cells after feeding. We subjected *Drosera* tentacles to high-pressure freezing (HPF, Moor 1987) followed by freeze substitution, a technique that had been shown to preserve delicate details in *Drosera* tentacle stalks, including notably cytoplasmic microtubules (MTs), actin microfilaments (MFs), and closely associated elements of endoplasmic reticulum (ER) (Lichtscheidl et al. 1990). We also analyzed the ultrastructure of the various glandular cell types during absorption and further processing of proteins, and relate the results to parallel observations of cells in the living state by fluorescence microscopy. We administered fluorescent bovine albumin (BSA) to *Drosera* leaves to mimic the chemical situation of the prey that is required for enzyme discharge and causes further secretion of digestive enzymes (Dexheimer 1978). We visualized this marker within the cells, and observed the fate and dynamics of the plasma membrane with the fluorescent membrane marker FM4-64.

## Material and methods

### Plant material

**Fig. 1:**
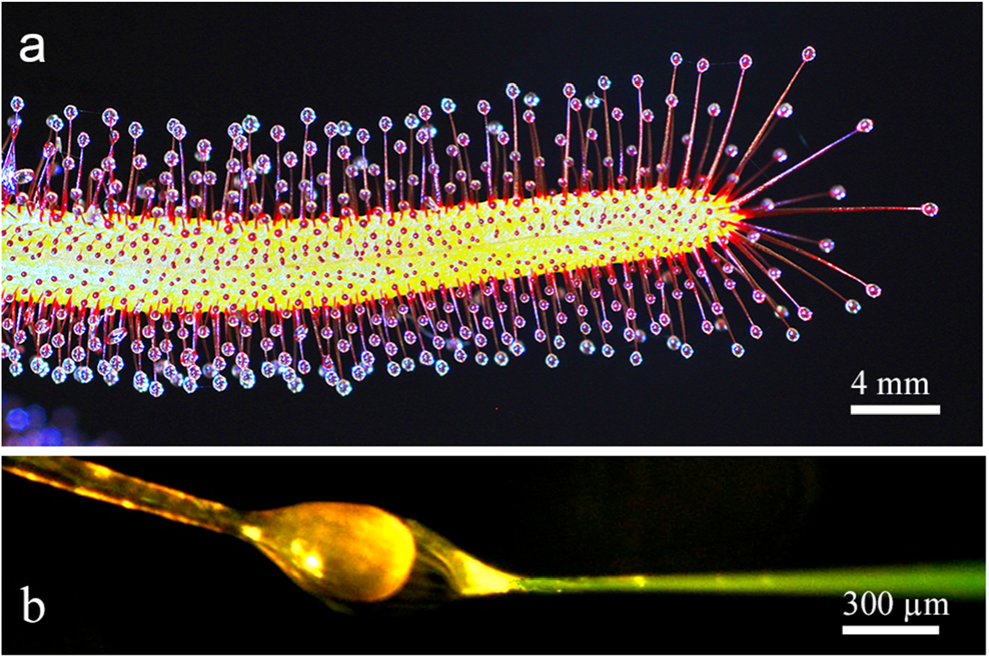
Leaf of *Drosera capensis*. Upper leaf surface and margin are covered with tentacles of different lengths that produce trapping mucilage (**a**). Nutrients and fluorescent indicator dyes are supplied to individual glands with the aid of a micropipette (**b**)

We investigated young but fully developed leaves of *Drosera capensis* that had been grown in the greenhouses of the Universities of Massachusetts and of Vienna without fertilizer in a mixture of sand and sphagnum. The long slender leaves bear tentacles the heads of which were each surrounded by a droplet of mucilage (Fig. 1a). According to the classification of Outenreath and Dauwalder (1982), they were in their intermediate to mature state. For our investigations, we used only healthy leaves that had had no contact with insects; we used them as such, or fed them for various times with BSA (5–10% in water) as a model for animal prey.

### Light microscopy

Leaves were studied macroscopically with a stereomicroscope (Nikon) equipped with a Nikon digital camera. This allowed us to feed individual tentacles with a glass capillary mounted on a micromanipulator. Dynamic properties of the glands and stalk cells were observed in a Leica DMIRE 2 and a Nikon Eclipse microscope both equipped with differential interference contrast (DIC) and epifluorescence. Chloroplasts were detected by their auto-fluorescence. The fluorescence of ER was observed by staining tentacles with 20 μM DiOC_6_ (3,3′-dihexyloxacarbocyanine iodide; Thermo Fisher) dissolved in water or in BSA. For fluorescence studies, in addition, we used a Leica DM 6000CS confocal laser scanning microscope. The tonal range adjustment of the micrographs and arrangement of plates was carried out in Adobe Photoshop CS6.

### Experiments on endocytosis

Uptake of proteins from animal prey was simulated by the soluble protein BSA. For fluorescence studies, 10 μl of 2% BSA coupled to FITC (FITC-BSA, Sigma) was deposited on the leaves for 5 min and up to 72 h, as described by Adlassnig et al. (2012). Thereafter, leaves were dissected and washed briefly in 2% BSA (Sigma) before observation. The conjugate made visible the passage of the protein into the gland and further into the tentacle. Alternatively, for localization of uptake in individual glands, FITC-BSA was loaded into a micropipette and placed over a single head with the aid of a micromanipulator (Fig. 1b).

As an alternative indicator for endocytosis, we marked the plasma membrane of the glandular cells with the styryl dye FM4-64 (N-(3-triethylammoniumpropyl)-4-(8-(4-(diethylamino)phenyl) (hexatrienyl) pyridinium-dibromide; Molecular Probes) at a final concentration of 4 μM either in water or in BSA in concentrations between 5 and 10%. With this probe, we could track the formation of endosomes (Jelinkova et al. 2019).

### Transmission electron microscopy by high-pressure freezing and freeze substitution

The central portion of the middle leaf zone bearing short tentacles at the surface was dissected and sandwiched as quickly as possible (20 s) in a mold between two lecithin-coated gold sample holders (Balzers BB1131242-1). For mechanical protection as well as for maximum heat conductivity, we filled the remaining space within the sample holder with a droplet of 1% ultralow gelling (< 15 °C) agarose (Type IX, Sigma Chemical Co.). Freezing of the tissue was accomplished with the Balzers HPM 010 high-pressure freezer.

Freeze substitution followed the procedure described by Lancelle et al. (1986, 1987) and employed by Lichtscheidl et al. (1990). It thus resembled the method applied by Kiss et al. (1990) and Staehelin et al. (1990). Briefly, cryoimmobilized material was transferred to acetone containing 2% osmium tetroxide at approximately − 80 °C and freeze substituted for 36–40 h, then brought to room temperature gradually over a period of 5–6 h. Before embedding in a mixture of Epon and Araldite, the tissue was transferred to methanol and stained in 5% uranyl acetate in methanol for 2 h, then brought back to acetone.

After staining with lead citrate, ultra-thin sections were observed in a Jeol 100 CX or a Zeiss 902 electron microscope operated at 80 kV.

## Results

### General morphology of the glandular heads

**Fig. 2:**
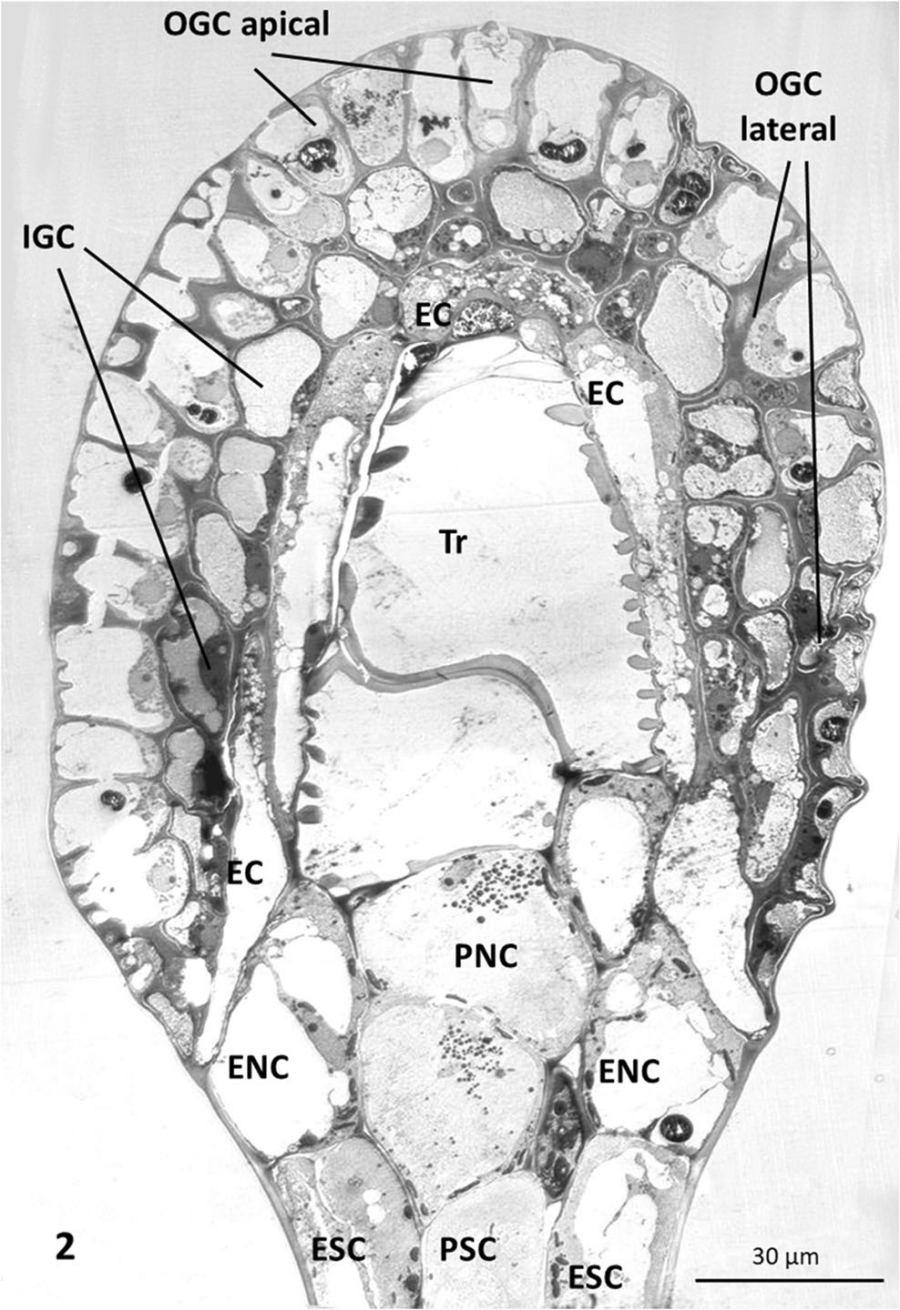
Overview of *Drosera* tentacles in TEM: the gland head is formed by an outer and inner layer of secretory cells (outer gland cells, OGC; inner gland cells, IGC). They are supported by a bell-shaped layer of endodermoid cells (EC) that curve out to the surface at the base of the head. They surround the core of tracheids (Tr) and connect to the stalk by a ring of epidermal and parenchymal neck cells (ENC; PNC). These neck cells are continuous with the cells of the tentacle stalk, the epidermal and parenchymal stalk cells (ESC; PSV). Transfer of substances from the glandular cells to the stalk is provided by plasmodesmata between glandular cells, endodermis, neck cells, and tentacle stalk

**Fig. 3:**
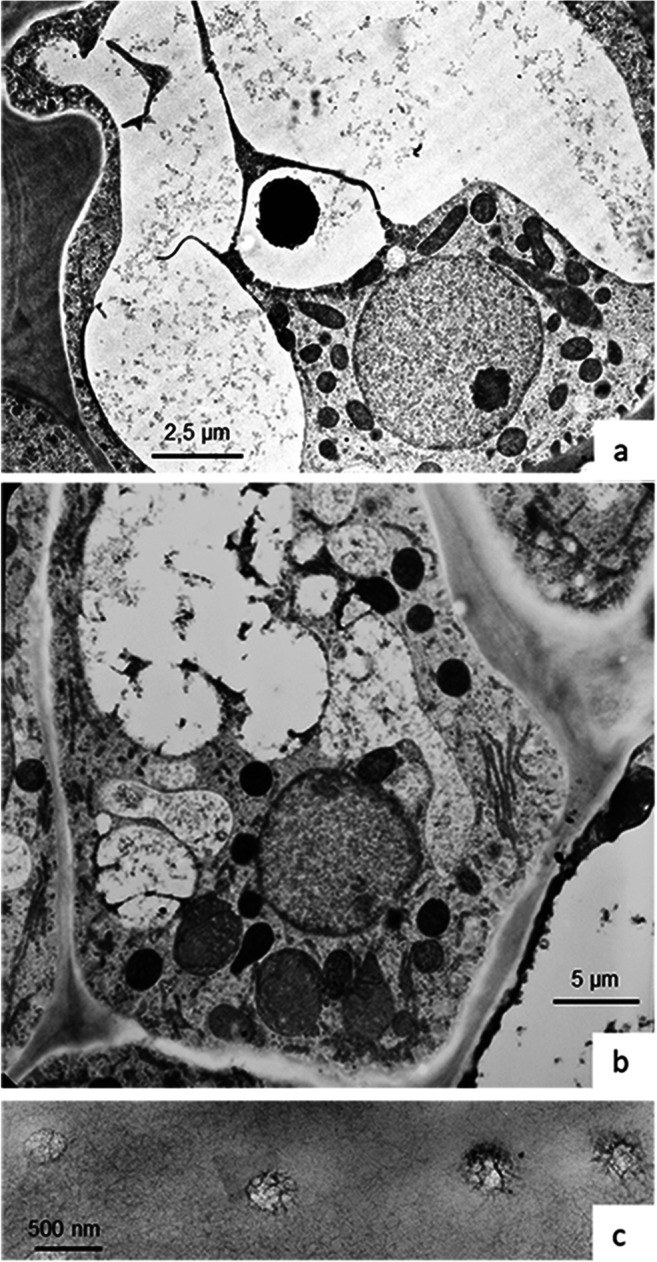
Outer (**a**) and inner (**b**) glandular cell. Cells contain a large vacuole surrounded by a layer of cytoplasm. The main part of the cytoplasm, including the nucleus and the organelles, is concentrated along the interior surfaces of the cell. The adjacent lateral walls as well as the peripheral tangential wall possess only a thin layer of cytoplasm with few organelles, mainly ER and vesicles. Leucoplasts with dense matrix and mitochondria are frequently found closely appressed to the nuclear envelope and in close contact with each other. Pores in the cuticle are filled with fluffy osmiophilic material (**c**)

Tentacles cover the upper surface and margins of the leaves. Their thin cylindrical stalk is fused into a glandular head by a connecting zone called the neck area, a ring of epidermal and parenchymal transfer cells. Glandular cells in the head produce trapping mucilage, a viscous solution of polysaccharides that can be drawn out into threads several centimeters long (Rost and Schauer 1977). The length of the tentacles varies and depends on their position on the leaf, with the marginal tentacles being up to ten times longer than the central ones (Fig. 1a). The cellular composition of the cylindrical glandular heads, however, is relatively constant, with the exception of some outermost tentacles carrying asymmetrical heads. Figure 2 shows two layers of glandular cells, the outer and inner gland cells; they cover the surface and are separated from a core of spongy tracheids with spiral-shaped thickenings by a bell-shaped layer of endodermoid cells. Their radial walls are impregnated with a waxy substance, presumably cutin or suberin, which inhibits apoplastic passage of small molecules between gland and stalk cells. The whole gland and also the stalk are covered by a cuticle. Pores with osmiophillic content are found only around secretory cells; these allow for passage of liquids (Fig. 3c).

Gland heads are connected to the tentacle stalks by a layer of cells described as transfer cells according to the definition of Gunning and Pate (1974), which were named as epidermal neck cells (ENC) and as parenchymal neck cells (PNC). They contain numerous plasmodesmata in their cell walls. Contact between tracheids of the gland head and the vascular supply of the leaf is given by a row of slender spiral tracheids that runs through the stalk. This xylem is surrounded by two rows of cells, an inner course of parenchymal and an external layer of epidermal cells. The different cell types are shown in Fig. 2.

### Ultrastructure of glandular cells before and after feeding with BSA

The periphery of the gland head is composed of two layers of closely fitting secretory cells. In this study, we only briefly summarize the composition of the cytoplasm and draw our focus on the endomembrane compartments involved in absorption, digestion, and transport of nutrients.

**Fig. 4:**
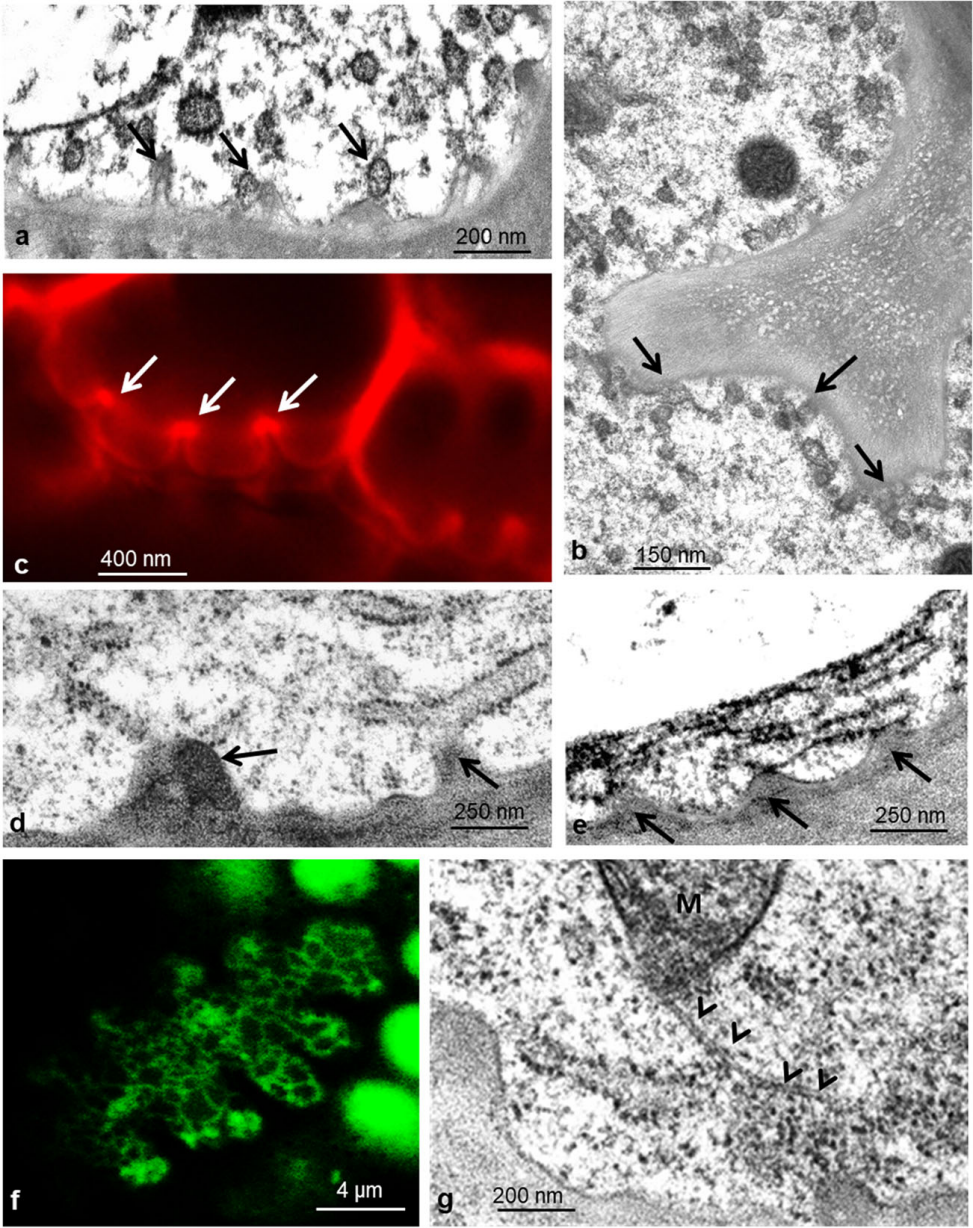
Outer glandular cell. Protuberances of the cell wall have different sizes and offer contact points for vesicles and ER. Contact between the plasma membrane and vesicles is seen in TEM (**a**, **b**) and after staining of the membrane with the fluorescent dye FM4-64 (**c**); arrows. Single ER cisternae are decorated with ribosomes and attached to the fingerlike invaginations of peripheral and lateral cell walls (**d**, **e**). Staining with DiOC_6_ shows the net of ER tubules in the peripheral cytoplasm of the outer tangential cell wall of gland cells (**f**). Occasional microtubules in the peripheral cytoplasm contact organelles, in this case a mitochondrion M (**g**); arrowhead

Outer and inner gland cells contain large eccentric vacuoles, which are traversed by strands of cytoplasm (Fig. 3a, b). Red pigment gives rise to osmiophilic content, presumably flavonoids and anthocyanins that are concentrated in electron-dense particles. Vacuoles are surrounded by cytoplasm that forms a thin layer underneath the outer peripheral cell walls where it contains mainly elements of ER and small vesicles. The main part of the cytoplasm occupies the interior part of the cells; here, the nucleus and most of the organelles are located. External and radial cell walls increase their surface by cellulose buttresses that form septa and in addition increase their surface with a fingerlike labyrinth of wall ingrowths, thus resembling the cell walls of transfer cells. They appear to be sites where vesicles make contact with the plasma membrane (PM, Fig. 4a, b) and endoplasmic reticulum (ER, Fig. 4d, e). Fluorescence labeling of ER with DiOC_6_ shows the three-dimensional arrangement of tubular ER in the outer periphery of the cytoplasm (Fig. 4f). Staining the plasma membrane with FM4-64 exhibits increased intensity where the cell wall protrudes into the cytoplasm and vesicles accumulate (Fig. 4c). The nucleus contains a large nucleolus and condensed chromatin concentrating inside the nuclear envelop. Leucoplasts with dense matrix and mitochondria are frequently found closely appressed to the nuclear envelope and in close contact with each other (Fig. 3).

Regarding elements of the cytoskeleton, we occasionally find straight or curved MTs mainly in the periphery of the cell (Fig. 4g), but also extending into the cytoplasm. By contrast, we have not observed actin MFs in either the outer or the inner gland cell. In agreement with this observation are those with the light microscope, showing that far-reaching organelle movements are not observed in these gland cells, only saltatory movement.

**Fig. 5:**
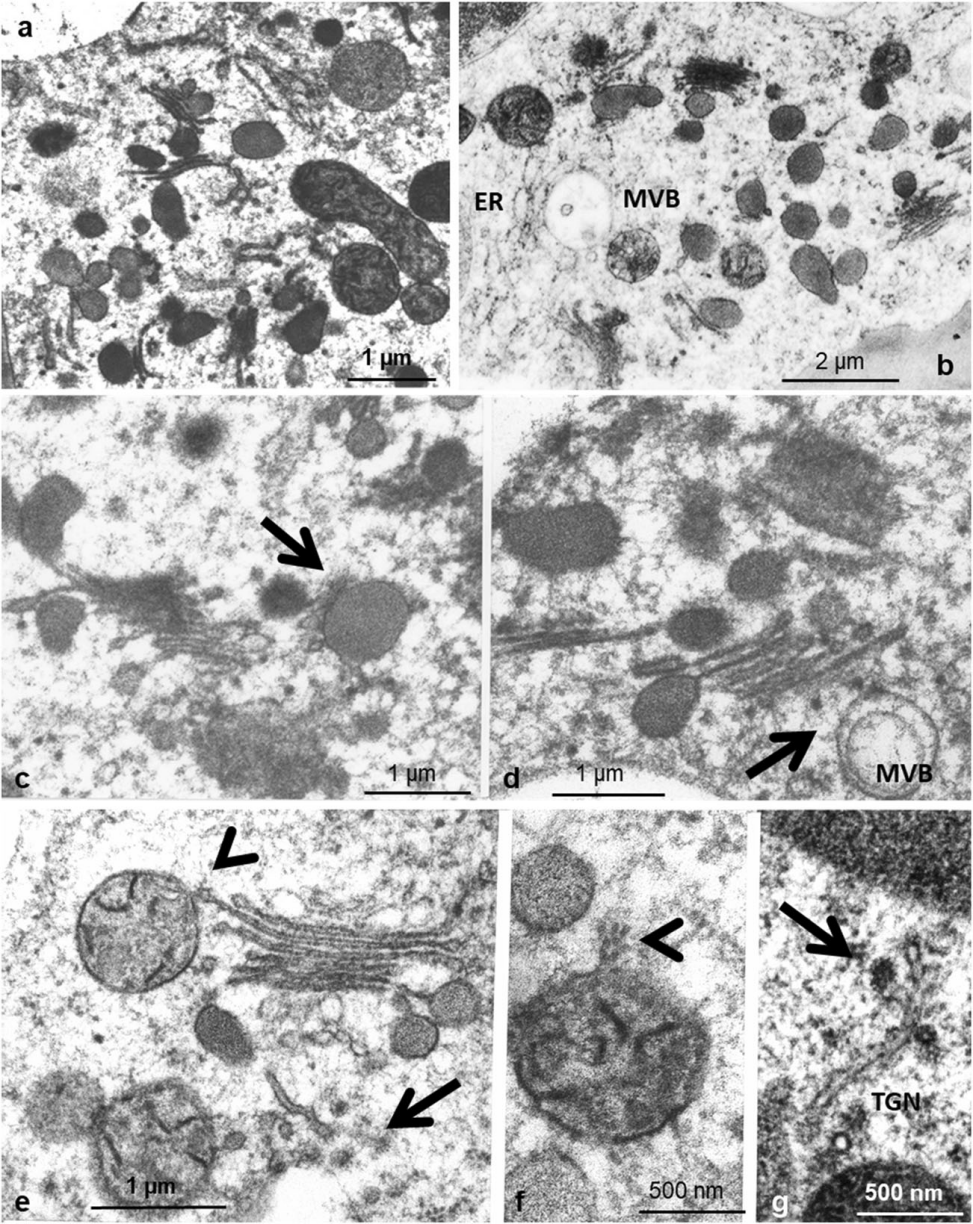
Golgi bodies in outer gland cells produce vesicles containing densely stained mucilage (**a**, **b**). In addition, the cytoplasm contains inflated cisternae of ER and small multivesicular bodies (MVB) resembling lysosomes (**b**). Small Golgi vesicles are in close contact with the mucilage containing microbodies (**c**) and with lysosomes (**d**); arrows. Membranes of the trans Golgi network (TGN) carry small vesicles bearing a proteinaceous coat resembling clathrin (**e**, **g**); arrows. Other vesicles bear a spiny coat and associate with Golgi cisternae and with organelles, especially with mitochondria (**e**, **f**); arrowheads

The ultrastructure of the endomembrane compartments such as ER, Golgi bodies, and microbodies depends on the physiological state of the tentacles. In unfed young tentacles, the most noteworthy feature of apical outer gland cells is well-developed Golgi bodies, which are responsible for slime production. They may consist of only a few lamellae, or may be multi-stacked, with straight or oblique arrangement of the lamellae (Fig. 5a–d). Golgi lamellae of the maturing face form dark-stained spherical or elongated slime vesicles of variable diameter with mostly homogenous electron-dense content. In addition, small smooth vesicles are attached to the Golgi; by coalescing they form large vesicles (Fig. 5c), and contact multivesicular bodies (Fig. 5d). Few small spiny vesicles are associated with Golgi lamellae of the forming face (Fig. 5e). Such vesicles associate not only with endomembranes, but also with organelles such as mitochondria (Fig. 5f). Membranes of the trans Golgi network (TGN) carry small vesicles bearing a proteinaceous coat resembling clathrin (Fig. 5g).

**Fig. 6:**
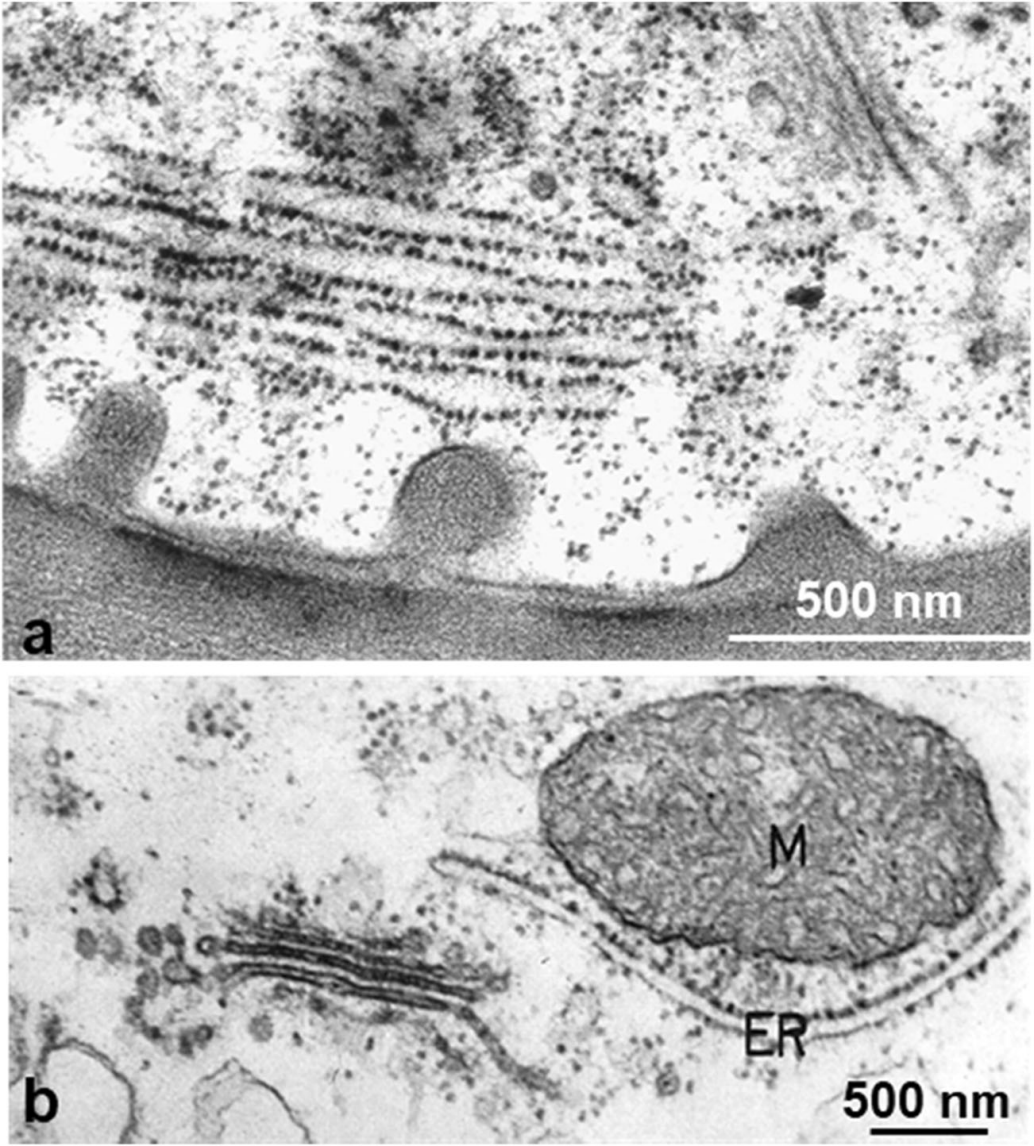
Gland cells after feeding with proteins. Cisternae of rough ER proliferate (**a**). Gradually the number of Golgi cisternae reduces and large Golgi vesicles disappear. Golgi stacks instead are in close contact with small vesicles which partly bear a coat of spiny proteins (**b**)

In glands of a later phase and after feeding with proteins, Golgi bodies look different; having terminated most of their mucilage production, they consist of only few narrow lamellae. Small vesicles are connected to the lamellae (Fig. 6b). Multivesicular bodies and vacuoles with residual bodies of various size and density appear. Due to the multitude of vesicles and Golgi/ER membranes in these cells and due to the static nature of the EM pictures, it becomes extremely difficult to connect the vesicles to their membrane source.

Elements of the ER appear mainly as singular cisternae in young cells and have no electron-dense content (Fig. 5b). In mature cells, when Golgi bodies cease slime production, and especially also after feeding, the smooth ER decreases while rough ER proliferates (Fig. 6). ER cisternae are arranged in long parallel stacks and as long profiles that follow along and are in close proximity with the PM mainly where the cell wall protrudes into the cytoplasm. In some instances, they give the appearance of being rigidly ordered by an underlying association with elements of the cytoskeleton.

### Absorption of fluorescent proteins by glandular cells

**Fig. 7:**
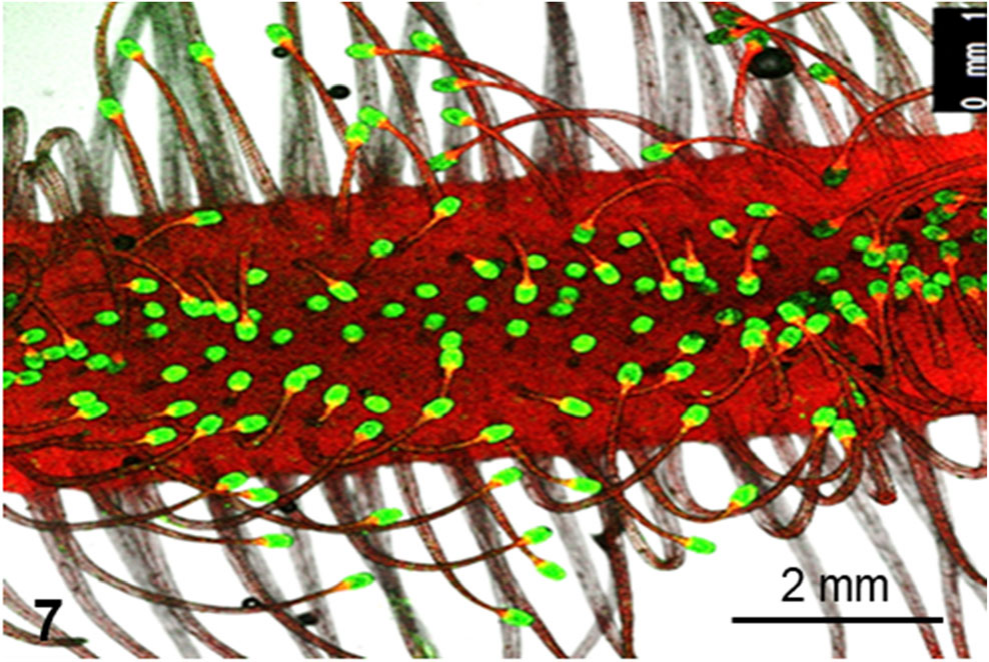
Leaf of *Drosera capensis* after feeding with FITC-BSA: the tentacles fold towards the center of the leaf. The glandular heads are brightly fluorescing in green because they absorbed the fluorescent protein. Chloroplasts in the leaf give red auto-fluorescence

Feeding with BSA results in a complex series of events including changes in leaf morphology as well as prominent changes at the subcellular level. For example, at a multicellular level, the application of a stimulus causes a folding of the tentacles locally towards the center of the leaf. While at the subcellular level, the application of BSA coupled to FITC results in the appearance of fluorescence signal in the glandular heads (Fig. 7).

**Fig. 8:**
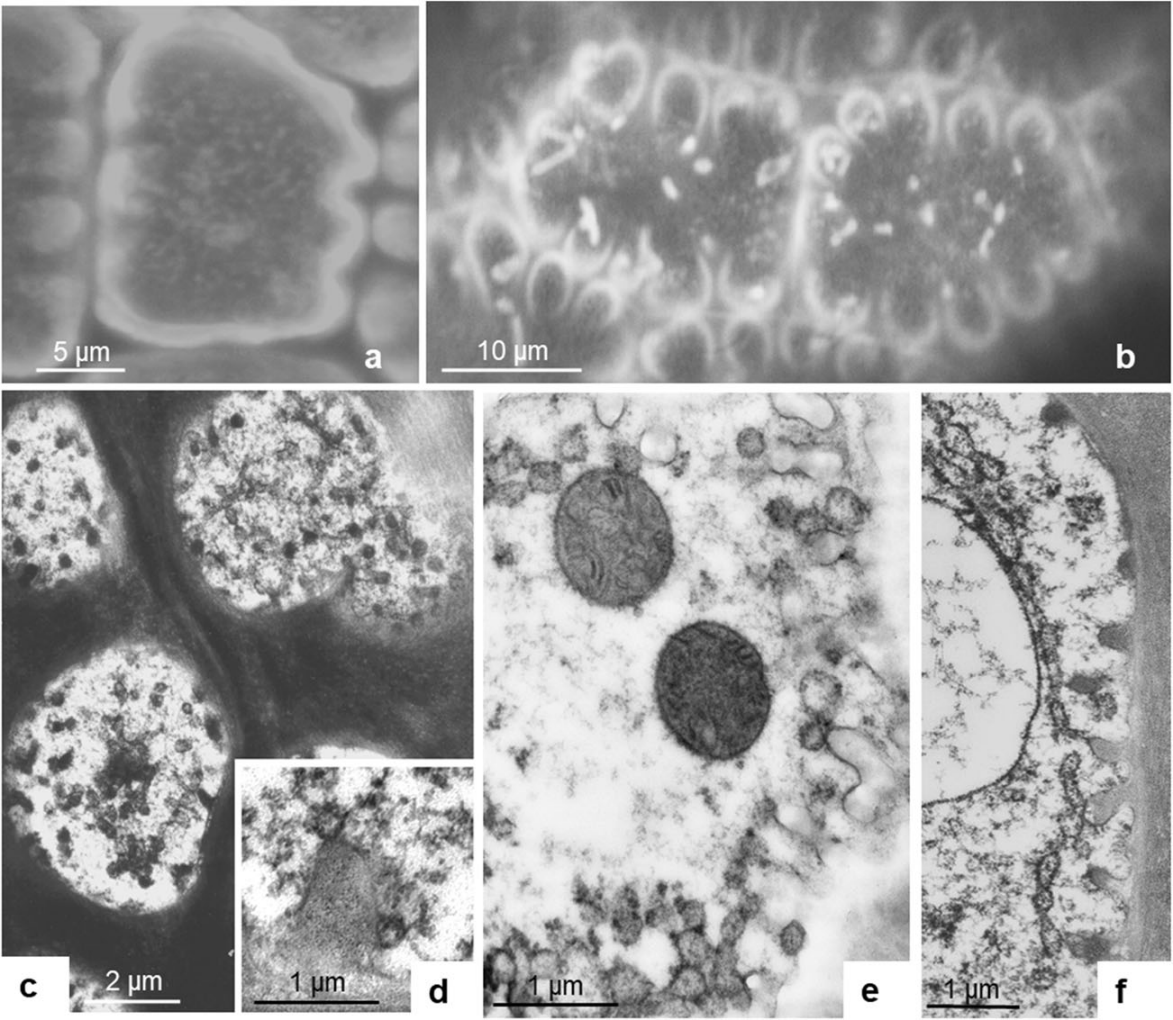
Staining of gland cells with FM4-64 shows membrane flow in the fluorescence microscope. **a**: Initially, the plasma membrane is decorated with fluorescence dots at the limit of resolution. **b**: larger vesicles of globular or tubular shape form. Spontaneous endocytosis occurs in untreated cells, but we observe it in higher frequency after feeding with BSA. **c**, **d**, **e**, **f**: EM sections show these vesicles closely attached to the plasma membrane and also deeper within the cytoplasm

**Fig. 9:**
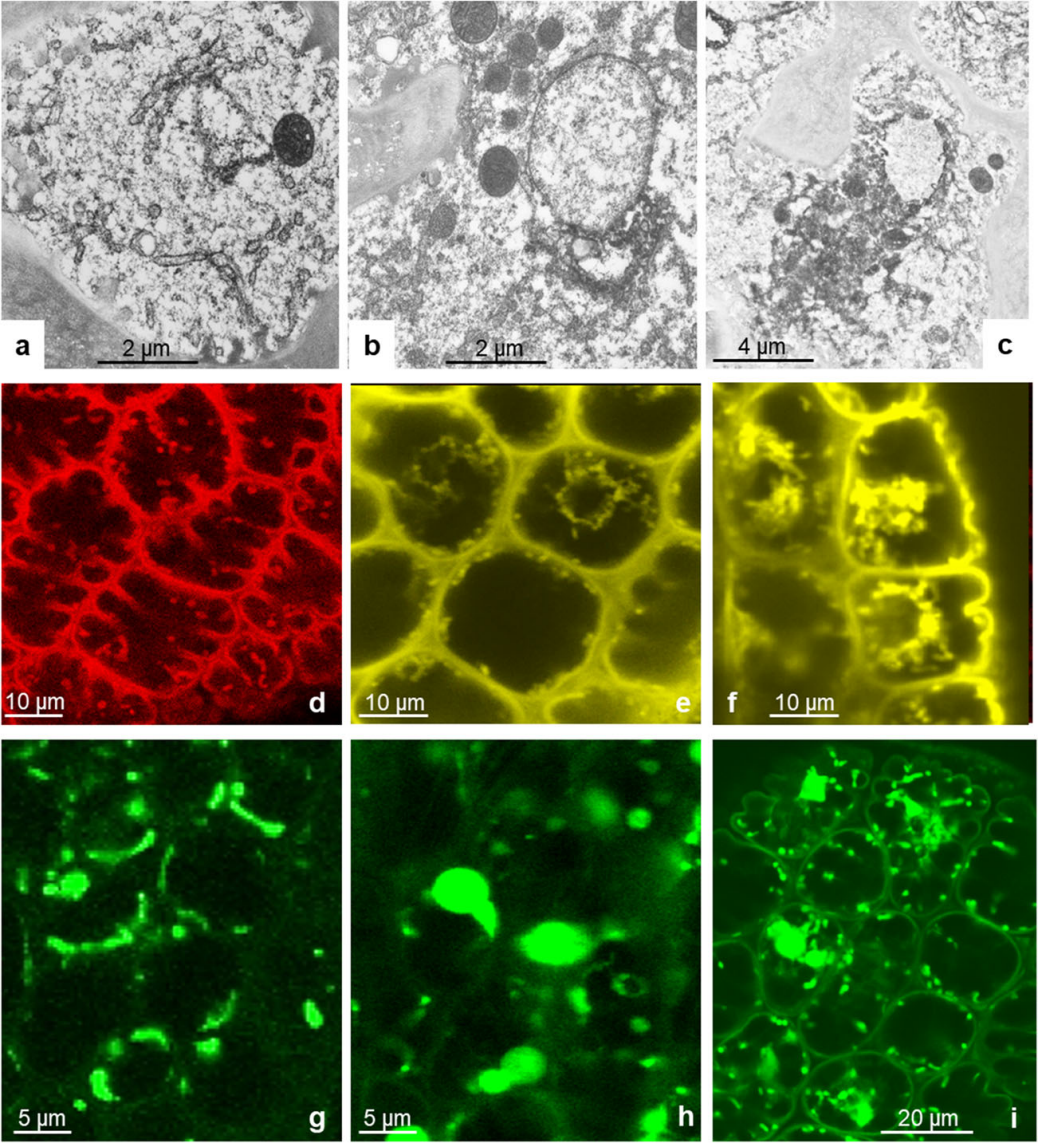
Outer gland cells in different levels of the cytoplasm during feeding with BSA: **a**, **d**, **g** peripheral cytoplasm in the surface of the gland; **b**, **c**, **e**, **f**, **h**, **i** central and inner side of the outer gland cells. TEM pictures show the tubulo-vesicular net of endosomes that form in the periphery; by fusing, they give rise to large vacuolar compartments (**a**, **b**, **c**). Staining of the plasma membrane with FM4-64 shows the fluorescent membranes of endosomes and their final destination into multivesicular bodies (**d**, **e**, **f**). By feeding with FITC-BSA, the absorbed proteins become visible within these endosomes (**g**, **h**, **i)**

**Fig. 10:**
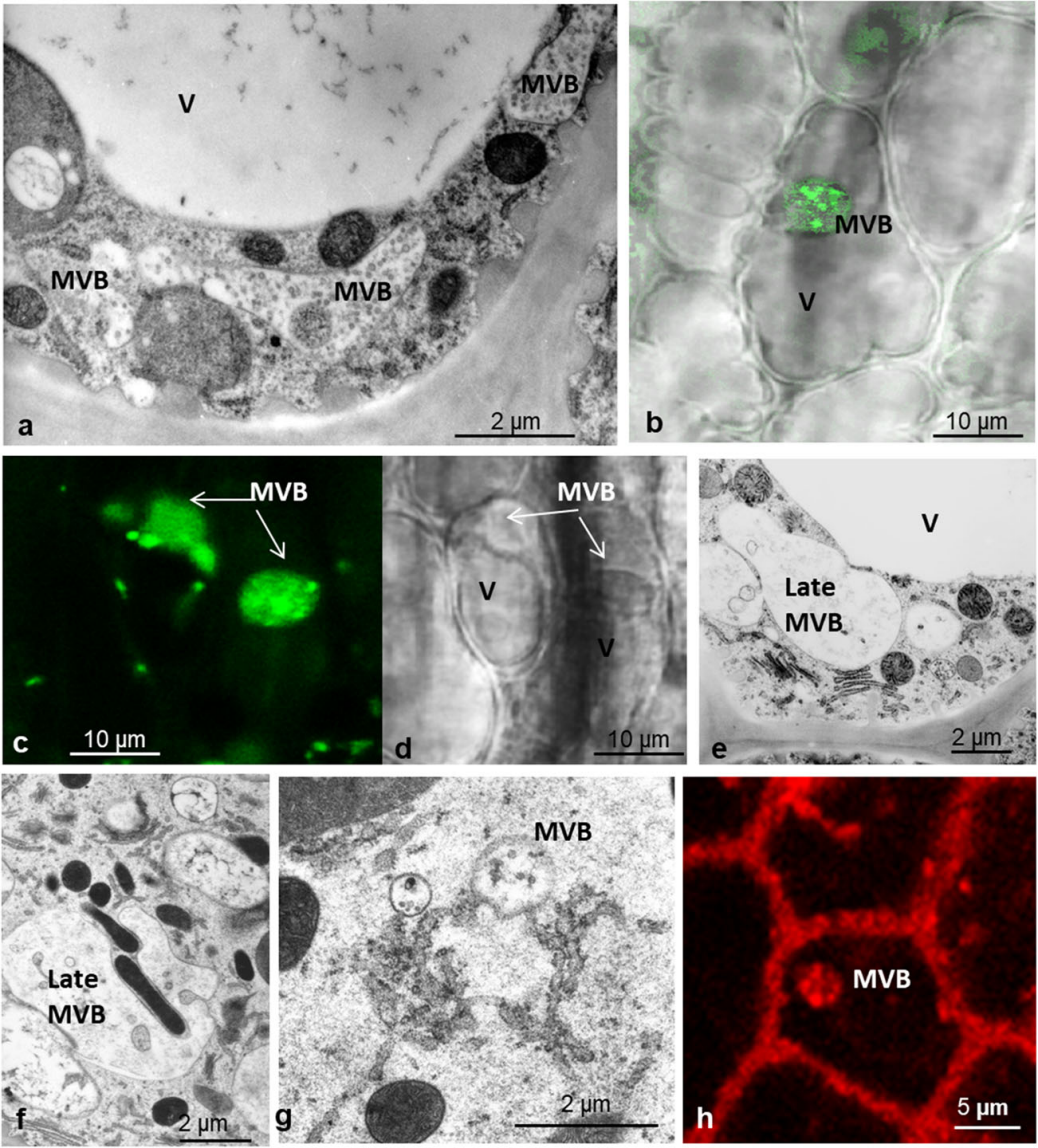
Multivesicular bodies (MVB) originate from endosomes. They fill a large portion of the cytoplasm of gland cells. In TEM pictures, the degradation of their content becomes visible: vesicles are still present (**a**), but gradually disappear (**b**). They feed on the tubulo-vesicular endosomes (**g**) and engulf also other organelles such as mitochondria (**f**). Absorption of FITC-BSA shows the content of endosomes of various sizes and MVBs with degrading substance (**b**, **c**). Comparison of such late endosomes in bright-field suggests that these compartments are different from the original cell sap vacuole (V) (**b**, **d**). Staining with FM4-64 shows their membranes in the fluorescence microscope (**h**)

The use of the fluorescent dye FM4-64 provides a clear demarcation of the plasma membrane and permits the analysis of endocytotic trafficking. The plasma membrane becomes punctuated with small dots at the limit of resolution (Fig. 8a, b), initially in the thin surface layer of the cytoplasm at the periphery of the outer gland cells and at anticlinal walls between outer gland cells (10–20 min incubation). Here, cells have the largest absorptive surface due to numerous invaginations of the cell wall. TEM images reveal these endosomes at the cell wall as well as their fusion products, chains of tubule-vesicular compartments (Fig. 8c–e). Time lapse showed the appearance of vesicular and tubular microbodies and a time-dependent increase of labeling inside enlarging clusters in the bulk cytoplasm as well as vesicles transformed into multivesicular bodies (Figs. 8f, 9, and 10). In a similar way, FITC-BSA becomes visible within small fluorescent vesicles, which gradually increase in number and size, and give rise to a new type of globular or elongated pleiomorphic vacuoles. These are independent of the original cell sap vacuoles and with time become heavily stained. In the EM, we see these endosomes as multivesicular bodies from variable size and structure with more or less residual content. Their content of small vesicles increases with time.

This uptake of FM4-64-labeled plasma membrane occurs in control conditions suggesting spontaneous endocytosis, but appears to be amplified in cells treated with BSA.

Further export of fluorescent BSA to the leaf was analyzed by pulse-labeling; we offered FITC-BSA to the leaves for 3–6 h, and then carefully washed it away. After 24 h, there was still intensive fluorescence of vesicular compartments mainly in the bulk cytoplasm similar to that seen also immediately after staining. In addition, some vesicles occurred in the cytoplasm of the neck cells and the tentacle stalk.

### Transport of proteins to the tentacle stalk

**Fig. 11:**
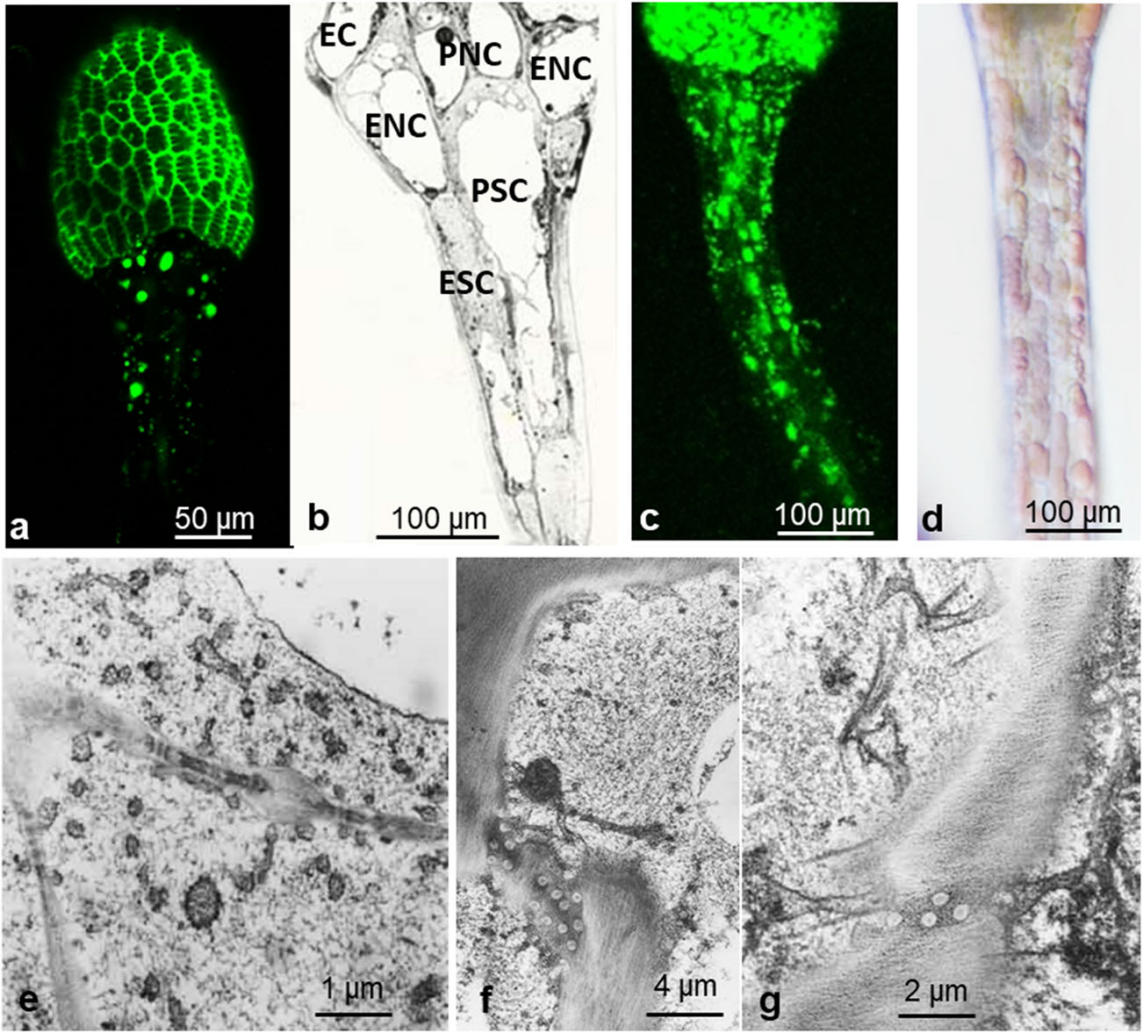
Distribution of FITC-BSA from gland head through neck cells to stalk cells. FITC-BSA occurs in endosomes of glandular cells (**a**) and further in epidermal and parenchymal cells of the stalk (**c**). In epidermal and parenchymal neck cells, it seems to accumulate within large round bodies (**a**). After feeding with BSA, the colorless cytoplasm of the stalk cells is swollen, whereas red vacuoles decreased in size (**d**; differential interference contrast). In BSA-treated stalk cells, the cytoplasm contains a large amount of small vesicles (**e**). A grazing section through neck and stalk in TEM shows the organization of the stalk cells (**b**). Plasmodesmata in transverse cell walls anchor ER (**f**, **g**). EC, endodermoid cell; ENC, epidermal neck cell; PNC, parenchymal neck cell; ESC, epidermal stalk cell; PSC, parenchymal stalk cell

For exploitation of absorbed proteins by the leaves, nutrients must be transported from outer gland cells to inner gland cells, and further to the endodermis. From there, these substances need to pass through epidermal and/or parenchymal neck cells to the tentacle stalk, and alternatively also transport through the central tracheid of the stalk was suggested (Figs. 2 and 11a). We analyzed the ultrastructure of the relevant cell types by EM and followed the path of FITC-BSA by fluorescence microscopy.

#### Endodermal cells

A layer of endodermal cells forms the “parenchymal bell” separating the inner gland cells from the tracheary elements in the center of the gland head. Rather short in the apical zone of the gland, lateral endodermal cells are elongated and flat, and they curve out to the surface at the base (Fig. 2; Suppl. Fig. 2a, b). Their radial cell walls are suberized similar to endodermal cells of roots, which contain a Casparian strip. The cells comprise a large central vacuole and a thin layer of cytoplasm with only few organelles including Golgi bodies and mitochondria. Very few plastids occur which, depending on the individual plants whose glands were sectioned, resembled either leucoplasts or chloroplasts. If no protein digestion takes place, the cytoplasm contains only few additional small vacuoles.

In these innermost living cells, during freeze fixation, ice crystal formation occurs more often than in the gland cells, but still we find sufficient amounts of well-frozen cytoplasm. Yet, the structure of the cytoplasm differs from that of the gland cells; it looks more loose and extracted. Bundles of MFs are closely coaligned with ER and oriented parallel to the longitudinal axis of the cell (Suppl. Fig. 2c, d). They are found deep within the cytoplasm, but approach the plasma membrane and cell wall as well. Accordingly, in video light microscopy, fast long-distance movements of organelles are observed.

#### The transfer cells of the neck region

The neck area completes the gland head and fuses it to the tentacle stalk. It consists of a peripheral cell layer of eight to ten cells forming a circle that make contact with the above out-curving endodermal cell and to the underneath positioned epidermal stalk cells (“epidermal neck cells”). A second layer within is a ring of parenchymal cells that connects to the above tracheids and endodermis of the glandular heads and impinges at its base on parenchymal cells of the tentacle stalk surrounding the central tracheid (Fig. 2 and 11b). Large numbers of plasmodesmata suggest symplastic connections between endodermis of the cells of the stalk (Fig. 11e–g). Both cell types have large central vacuoles surrounded by a thin layer of cytoplasm containing an often tapered nucleus and chloroplasts in addition to the usual organelles. Dark osmiophilic precipitates in the vacuoles represent most probably the red anthocyanin known in these cells from light microscopy. In addition, extensively stained homogenous round bodies cluster in the vacuole of parenchymal neck cells (Suppl. Fig. 3d, e). They have strong auto-fluorescence with a maximum emission between 540 and 570 nm, and are in constant Brownian motion (Suppl. Fig. 3a, b). They continue also in the neighboring parenchymal cells of the stalk in its upper region and are independent of protein supply in both unstimulated and BSA-treated cells.

The preservation of the cytoplasm in both cell types appears to be quite good (Suppl. Figs. 4; 5): the nucleoplasm has a fine, granular texture with homogenous nucleoli and a smooth nuclear envelope. The somewhat extracted appearance of the cytosol is similar to that of endodermal cells. Organelles of both epidermal and parenchymal neck cells often make close contact, and this is also the situation in the adjacent epidermal and parenchymal cells of the tentacle stalk: mitochondria and chloroplasts tightly appress to nucleus and microbodies; mitochondria are attached to chloroplasts and even penetrate them (Fig. 13g; Suppl. Fig. 5a, b, c). In addition to intruding organelles, inclusions of cytosol and extensions resembling stromules are observed in the pleiomorphic chloroplasts (Fig. 13h, i; Suppl. Fig. 5c)

#### Epidermal and parenchymal cells of the stalk

**Fig. 12:**
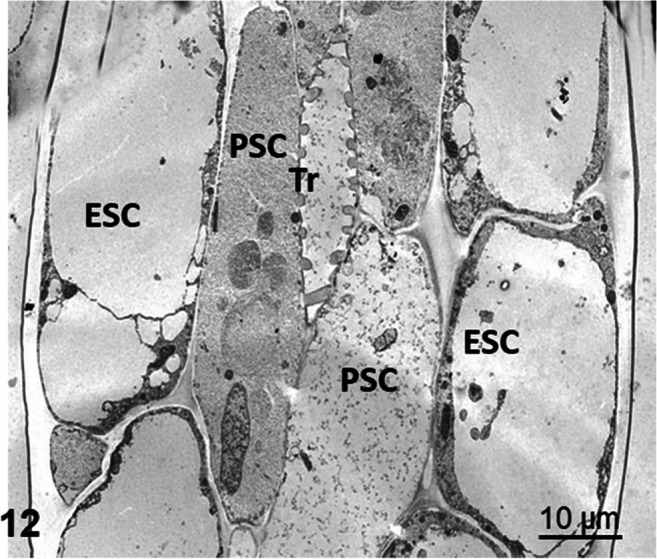
Cross section through a tentacle stalk of *Drosera capensis*. A central tracheid (Tr) is surrounded by an inner ring of long parenchymal stalk cells (PSC) and an outer ring of shorter epidermal stalk cells (ESC)

Similar to the neck cells, a ring of parenchymal cells ensheathes the central spiral tracheid in the stalk. It is surrounded by a second ring of cells forming the epidermis (Fig. 12). The epidermis carries occasional glandular trichomes consisting of 2 to 6 glandular cells (not shown).

Between endodermis and neck cells as well as in the transverse walls between the stalk cells, plasmodesmata group into pit fields (Fig. 11e–g). In the adjacent cytoplasm, vesicles of different size and complexity accumulate in clusters (Fig. 11e). Feeding leaves with proteins causes re-organization of the whole cytoplasm, a complex reaction described much earlier as “aggregation” (Darwin 1875). The cytoplasm starts to swell immediately after feeding, the velocity of organelles increases, and the cell sap vacuole segregates into many small parts that move through the cell (Fig. 11d). While vacuoles and most organelles accelerate their movement and are in constant cyclosis, chloroplasts remain stably anchored to the cell wall, mainly along the inner longitudinal wall of epidermal and parenchymal stalk cells.

Some vacuoles contain spherical inclusions similar to those found in the parenchymal stalk cells. Also, some vacuoles exhibit strong auto-fluorescence with similar spectral characteristics as those spherical inclusions found in the neck cells, and which also occur in the vacuoles of a few stalk cells (Suppl. Fig. 3c).

**Fig. 13:**
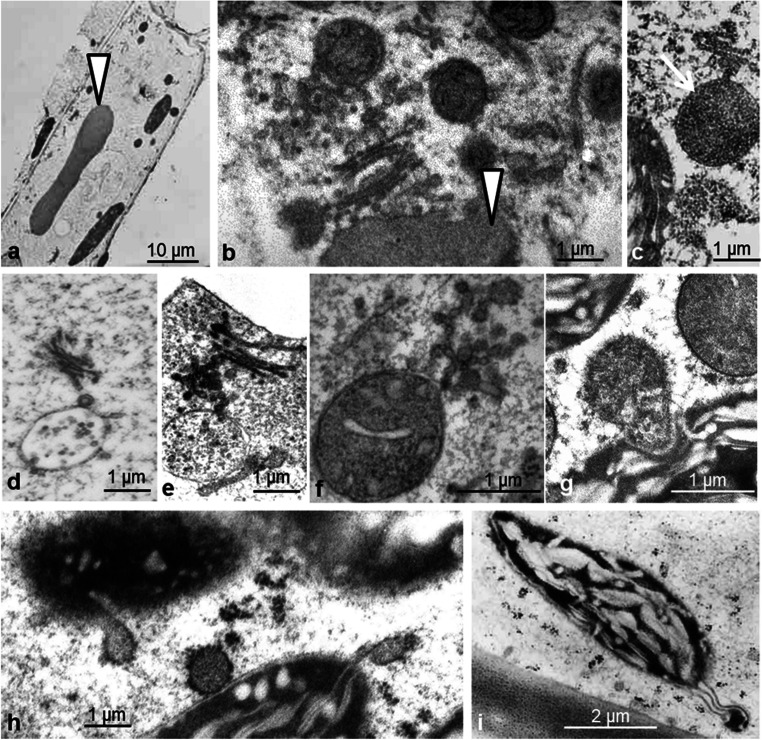
Organelles and protein bodies in epidermal and parenchymal cells of tentacle stalks. Protein bodies, earlier described as rhabdoids, occur in untreated and in BSA-treated cells (**a**, **b**). Golgi vesicles connect with rhabdoids (**b**, **c**). Golgi vesicles also connect to multivesicular bodies (**d**, **e**) and to mitochondria (**f**). Organelles such as mitochondria and chloroplasts closely interconnect (**g**). Chloroplasts interconnect with ER and form stromules (**h**, **i**)

Rhabdoids, elongated protein bodies, are present in the epidermal cells of untreated leaves and occur in BSA-treated cells as well. They are in close contact with Golgi vesicles. From these static pictures, we cannot say if proteins are added to or consumed from the protein bodies (Fig. 13a, b).

#### Progress of FITC-BSA from glandular heads to stalk

Feeding leaves with FITC-BSA leads to the formation of fluorescent endosomes in the endodermis after 1 to 2 h; after 24 h, these endosomes are observed in the parenchymal and epidermal neck and further in the living cells of the tentacle stalk, mainly in the epidermis. Large amounts of FITC-BSA cause fluorescence of the cytosol of living stalk cells in addition to fluorescent organelles. The central tracheids of the gland head and stalk occasionally showed fluorescence in young not fully developed leaves.

#### Progress of vacuolar staining by DiOC_6_ from glandular heads to stalk

In an attempt to stain ER and mitochondria, we applied DiOC_6_, a fluorescent indicator for membrane potential. It successfully allowed observation of ER and mitochondria in outer and inner gland cells (Fig. 4f), but in addition, it gradually stained the cell sap of the vacuoles, clearly showing the close contacts of outer and inner gland cells with the endodermoid cells (Suppl. Fig. 6a). This dual staining also extended into the tentacle stalk where it mainly occurred in epidermal stalk cells (Suppl. Fig. 6b). Some notable characteristics included a stable cortical net of ER that stained strongly; fast-moving endoplasmic ER tubules, which gave a diffuse fluorescence to the cytoplasm, where mitochondria shone brightly (not shown); and the vacuoles, which were heavily labeled. In control cells, the vacuoles were uniform and filled the cells (Suppl. Fig. 6c), whereas in BSA-treated cells, they were disintegrated into tubes and vesicles, which moved quickly through the swollen cytoplasm (Suppl. Fig. 6d).

## Discussion

In this study, we provide new information about the dynamic properties of gland cells of carnivorous plants when stimulated by exogeneously applied protein. Using both live cell and fixed preparations, we followed the absorption of proteins into gland cells of *Drosera capensis*, as well as the ensuing progress of nutrients from gland cells to tentacle stalk, including the “aggregation” of cytoplasm in stalk cells during feeding. In this phase, we used fluorescence staining as a means to provide information about the underlying physiological processes. In the studies of fixed cells, we took advantage of the high fidelity and extent of preservation in cells prepared by high-pressure freeze fixation and freeze substitution, and by examination at high resolution in the transmission electron microscope. By relating the observations from both techniques, a new look at dynamic events and structural properties has become possible.

### Absorption of proteins by endocytosis

Stimulation of gland cells through the application of proteins (FITC-BSA) led to the proliferation of elements of rough ER; this stood in contrast to unstimulated gland cells that had mainly smooth ER and only few cisternae lined by ribosomes. In addition, prominent associations and apparent connections were observed between these rough ER cisternae and the PM at sites of cell wall invaginations. These observations strengthen the suggestions of Heslop-Harrison and Heslop-Harrison (1981; reviewed by Juniper et al. (1989)) that digestive enzymes are produced in the ER cisternae and might be transferred directly from ER across the PM to the apoplast.

Application of FITC-BSA led to an immediate bending reaction of the tentacles within the first 10 min. During this interval, fluorescent vesicles formed on the plasma membrane in the outer periphery and the lateral cell walls of gland cells. They pinch off mainly from the fingerlike invaginations of the cell wall in the process of endocytosis. These observations support the report of Adlassnig et al. (2012) concerning endocytosis in several glands of carnivorous plants including *Drosera*. They clearly underline the findings of Baluska et al. (2004), Samaj et al. (2004), Exteberria et al. (2009), Exteberria (2012), and recently Narasimhan et al. (2020) that fluid-phase endocytosis is a primary route for the exchange of solutes between the apoplast and cytoplasm, and the further trafficking of endomembranes as was summarized, e.g., by Hu et al. *(*2020). Clathrin-mediated endocytosis similar to animal cells was discussed also for plant cells, and indeed, we did find vesicles in the cytoplasm bearing proteins structurally similar to clathrin (Fig. 5).

A question remained if endocytosis of early endosomes was triggered by the BSA fused to the fluorescent dye, and we therefore also offered the fluorescent styryl dye FM4-64, a well-established membrane marker for endocytosis (Jelinkova et al*.* 2019), together with and without a stimulating protein. In this instance, the plasma membrane also became decorated by fluorescent dots, close to the limit of resolution, which pinched off and fused to form larger aggregates. These observations suggest that endocytosis is not necessarily dependent on the presence of nutrients in the apoplast, but that spontaneous membrane invaginations and endocytosis occur as well. A possible explanation could be that in gland cells, which have the main function of secreting trapping mucilage and digestive enzymes, the endocytotic machinery is active for membrane retrieval after excessive secretion.

Outenreath and Dauwalder (1986) showed the incorporation of tritiated (3H)-galactose into tentacles of *Drosera capensis*, and from their results, it appeared that the outer gland cells are not uniform in the activity; thus, radioactive material accumulated in apical rather than in radial outer gland cells and the apical cells were also more active in secreting trapping mucilage than the lateral cells (Juniper et al*.* 1989; Ellison and Adamec 2018). In the experiments presented here regarding absorption by *Drosera capensis*, we could not find such a difference between lateral and apical gland cells, as they both had similar staining of the PM with FM4-64 and endocytosis with FITC-BSA; we cannot exclude, however, that such micro-morphological features could depend on the age of the leaves or on the position of the tentacles on either center (short tentacles) or margin (long tentacles) of the leaves, or on various *Drosera* species.

Fluorescent vesicles fuse to form larger structures, similar to the vesiculo-tubular complexes observed in the pictures from EM (Figs. 8 and 9). These give rise to multivesicular bodies and to a set of large complex compartments seen in both fluorescence and in the EM. In live cell imaging, these new organelles can be well discriminated from the original cell sap vacuoles because of the red anthocyanins in the original cell sap. Thus, they are independent of the original cell sap vacuole and moreover form de novo. This could be a reason for the swelling of the cytoplasm described as “aggregation” (Darwin 1875), while the original vacuoles decrease in size.

### Translocation of absorbed products to tentacles

Transport of absorbed substances from the glandular head through the tentacle stalk to the leaf has been proven, but the route that the substances take is still not clear. The large number of plasmodesmata in cell walls between gland cells, endodermoid cells, neck cells, and the two layers of living cells in the stalk support a mechanism of symplastic transport (Williams and Pickard 1974). On the other hand, Gilchrist and Juniper (1974) found blebs in the endodermoid cells, which evaginated towards the spongy tracheid mass in the center of the gland head and might suggest movement through the xylem.

Absorbed FITC-BSA distributed within one hour between all cells of the gland including the endodermoid cell, but we did not observe it in the tracheid center of the gland head. Further transport through transfer cells in the neck gave rise to fluorescent vacuoles and vesicles in the cytoplasm of the living stalk cells, mainly the epidermis. However, neither the cytoplasm *per se* nor the vacuoles were fluorescent, and similarly, the tracheids in the stalk were not fluorescent either. Despite the progress of fluorescence to the stalk, the glandular cells in the head remained fluorescent, suggesting that not all proteins are necessarily exported. In EM pictures, we see vesicles of different sizes distributed within the whole swollen cytoplasm, but also accumulating around plasmodesmata in transfer cells, epidermal stalk cells, and parenchymal stalk cells. These observations support the symplastic continuity of cells in *Drosera* tentacles.

A similar symplastic movement can be assumed for the ER stain DiOC_6_. In addition to the plasma membrane and ER (depending on concentration), it also stained mitochondria in the gland cell of the head, and gradually the mitochondria in the transfer cells of the neck and in the stalk cells. However, for reasons unknown to us, the vacuoles of the gland cells stained intensively after some time. We therefore presume continuity through plasmodesmata not only of the cytoplasm but also of vacuoles. In the EM, we never saw vacuoles in direct contact with plasmodesmata, but ER connects to plasmodesmata and could mediate the transport.

### Lessons learned from EM and quality of the fixation

Good-quality fixation is essential for EM studies in order to provide reliable information. In *Drosera* tentacles, chemical fixation is a problem because of the thick cuticle lining the stalk: fixatives such as formaldehyde and glutaraldehyde penetrate so slowly that substantial rearrangements of the cytoplasm occur during the fixation process (our own observations). Cryo-fixation circumvents this problem and preserves the cytoplasm in a fraction of a second; however, flawless vitrification is difficult to achieve except for the outermost micrometers (e.g., 10 μm) of tissues; in deeper layers of cells and tissues, ice crystal damage occurs. The technique of high-pressure freeze fixation followed by freeze substitution greatly increases the depth and extent of high-quality fixation as described by Knoll et al. (1987), Moor (1987), Studer et al. (2001), and McDonald et al. (2007) and since then yielded some excellent results in plant cells (e.g., Donohoe et al. 2007; Wilson and Bacic 2012; Karahara and Kang 2014; Gergely et al. 2018) and animal cells (e.g., Hess et al. 2018).

In *Drosera* tentacles, many cells were well preserved, despite the thickness of the cuticle and tissue (up to 100 μm). Ice crystal damage was found in few cells or parts of cells; it was not necessarily confined to inner zones of the tissue, although it happened there more frequently. Due to unaccounted differences in staining, we found that neighboring cells, which look very much alike, may be contrasted either smoothly or appear very dark and overstained. Unrelated to the quality of the cytoplasmic preparation, we found cracks and breaks in cell walls and cytoplasm that mainly appear in the periphery of the tissue, but may occur within as well. In addition, we occasionally found burst and ruptured nuclei. This may be due either to mechanical damage during high-pressure freezing (Kaeser et al. 1989; Kiss et al. 1990) or to conversion of Ice II or III to Ice I during substitution as was suggested by M. Mueller (ETH Zuerich, pers. communication) and Craig and Staehelin (1988); the latter is less dense, and thus, the conversion is accompanied by a volume expansion that may break the cell wall. In general, we gained the impression that the frozen material becomes more brittle and inelastic than chemically fixed material.

The form of plastids and mitochondria is different to many published pictures from chemical fixation: thus, after freeze fixation, mitochondria are branched and interconnected, and also chloroplasts are interconnected and show stromules that had been described earlier, e.g., by Natesan et al. (2005), Holzinger et al. (2008), and Hanson and Hines (2017). Thus, the rapid preservation achieved during freezing may prevent changes of organelle morphology.

Elements of the cytoskeleton such as MFs and MTs as well as ER were visible, and interrelation between them as well as with other organelles could be very well studied (see Lichtscheidl et al. (1990). In addition, in all cell types, organelles and ER are in close contact with each other; indeed, we rarely observe single individual organelles when we look at serial sections but we see complex associations between organelles of the same sort, e.g., plastids, and between organelles of different kinds, e.g., plastids, mitochondria, and the nucleus, and the ER is closely aligning and surrounding all (e.g., Volland et al. 2012). Close connections between organelles have been reported from other plant cells (e.g., Brown et al. 1983, reviewed, e.g., by Douce 1985), but compared to the large number of investigations with chemically fixed plant cells, these reports are rather few. Concerning endomembrane compartments, Mersey and McCully (1978) and McCully and Canny (1985) showed that the ER belongs to the most labile components of plant cytoplasm and undergoes drastic changes during chemical fixation that is avoided by freeze fixation providing a life-like structure of the cytoplasm. Strengthened by the good preservation of highly labile MFs, we therefore expect that the close interactions of membrane systems and organelles seen in *Drosera* after HPF-FS represent the situation as it occurs in living cells. The importance of contacts between ER and organelles has been shown recently in animal cells (Wu et al. 2018) and plant cells (Izumi and Nakamura 2018) and was reviewed for plant cells by Ye et al. (2020). We predict that HPF-FS will be a suitable technique for further research, especially when combining with newly developed techniques of accelerated freeze substitution (Reipert et al*.* 2018).

## Conclusions

In this study, absorption and distribution of proteins by glands of *Drosera capensis* were studied by administering the fluorescently labeled protein FITC-BSA and by analyzing the ultrastructure of the cells in EM after high-pressure freezing and freeze fixation. Fluorescent proteins are absorbed by gland cells through endocytosis. Endosomes fuse and form special vacuoles different from the cell sap vacuole. Membrane staining with FM4-64 shows that this is an autonomous process and not necessarily triggered by the presence of proteins. Fluorescent proteins progress from glands through neck cells to epidermal and parenchymal tentacle cells in the form of vesicles within the cytoplasm. Large amounts of proteins lead to additional staining of the cytoplasm. These observations indicate that plasmodesmata provide a symplastic route through which transport is possible. HPF-FS further reveals the close interactions of organelles, ER and the cytoskeleton during these dynamic events of protein absorption.

## Supplementary Information

Suppl Fig. 1 The cytoplasm fits into bays between buttresses of cell walls. **a**: EM sections show vesicles budding away from the PM and forming a tubulo-vesicular net that reaches into the cytoplasm and fuses with vacuoles. **b**: Staining of the PM with FM4-64 allows for *in vivo* imaging of the cells (PNG 1181 kb)

High Resolution (TIF 2695 kb)

Suppl Fig. 2 Endodermis of the glandular head. Endodermoid cells are short in the apical zone of the gland head (**a**) and flat and elongated along the flanks (**b**). They curve out at the base of the gland head. The radial walls between endodermal cells and the walls bordering the tracheids of the core are impregnated (arrows). The cytoplasm is well preserved despite the deep distance for freezing, and bundles of actin microfilaments account for the high mobility of organelles observed in live cell imaging (**c**, **d**) (PNG 1013 kb)

High Resolution (TIF 2327 kb)

Suppl Fig. 3 Neck region of *Drosera* tentacles. Homogenous round bodies cluster in the vacuoles of parenchymal neck cells (PNC) and parenchymal stalk cells (PSC), but not in the vacuoles of epidermal neck cells (ENC) and epidermal stalk cells (ESC). They move in Brownian motion, as seen in bright-field microscopy (**a)** and emit green fluorescence; chloroplasts shine in red (**b**). In sections from TEM, they reveal their osmiophilic nature (**d**, **e**). Occasionally also the vacuoles of parenchymal stalk cells emit diffuse green fluorescence with a similar spectrum as the globular structures (**c**) (PNG 2624 kb)

High Resolution (TIF 6519 kb)

Suppl Fig. 4 Epidermal cells of the neck and the stalk of *Drosera* tentacles. Organelle morphology is well preserved after High-Pressure Freezing. These organelles are in close contact with each other. In BSA-treated cells, the large central vacuole is disintegrated into several parts (**a**). Fixation and substitution cause dark osmiophilic precipitations. Chloroplast (C); Mitochondrion (M); Nucleus (N); Nucleolus (Nu); Cell sap vacuole (V) (PNG 2979 kb)

High Resolution (TIF 6343 kb)

Suppl Fig. 5 Organelle associations captured by freeze fixation. **a**, **b**, **c**: Chloroplasts (C) are in intimate contact with mitochondria (M); Pleiomorphic chloroplasts engulf portions of cytoplasm (asterisk); (**c**) (PNG 2941 kb)

High Resolution (TIF 8783 kb)

Suppl Fig. 6 Staining of *Drosera* tentacles with DiOC_6_ results in labeling of the cell sap vacuoles, in addition to mitochondria. In the gland head, outer and inner gland cells as well as endodermoid cells are clearly depicted (**a**). The labeling progresses to the stalk along epidermal stalk cells (**b**). In the epidermis, round and elongated mitochondria (arrows) possess fluorescence in an otherwise unstained cytoplasm, together with the vacuole (V). In untreated cells, the vacuole fills the cell (**c**), whereas in BSA-treated cells the vacuole is disintegrated into tubes and vesicles shining brightly within the swollen unstained cytoplasm (**d**) (PNG 1502 kb)

High Resolution (TIF 2923 kb)

## Data Availability

Data supporting the results reported in the article can be found in the Core Facility of Cell Imaging and Ultrastructure Research, University of Vienna, Althanstrasse 14, A-1090 Vienna, Austria All data generated or analysed during this study are included in this published article.

## Declarations

All authors agree with the following statements

The work described has not been published before; that it is not under consideration for publication anywhere else;

### Data transparency

All data and materials as well as software application support our published claims comply with field standards
