# Supplementary figures and images for: Gland cell responses to feeding in Drosera capensis, a carnivorous plant
Source: Protoplasma. 2021 Jun 21;258(6):1291–306. doi: 10.1007/s00709-021-01667-5 (PMC8523503; doi:10.1007/s00709-021-01667-5)

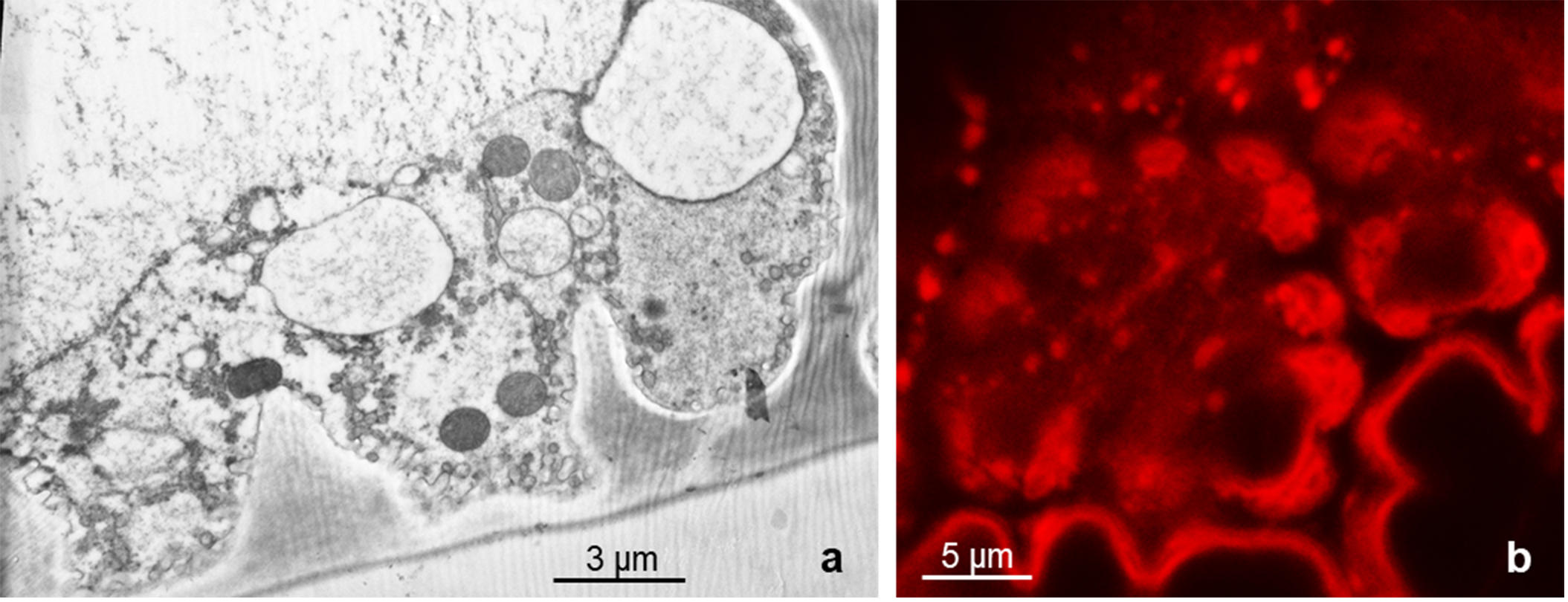

Supplement: Supplementary file 1 — The cytoplasm fits into bays between buttresses of cell walls. a: EM sections show vesicles budding away from the PM and forming a tubulo-vesicular net that reaches into the cytoplasm and fuses with vacuoles. b: Staining of the PM with FM4-64 allows for in vivo imaging of the cells (PNG 1181 kb) [file 709_2021_1667_Fig1_ESM.png]

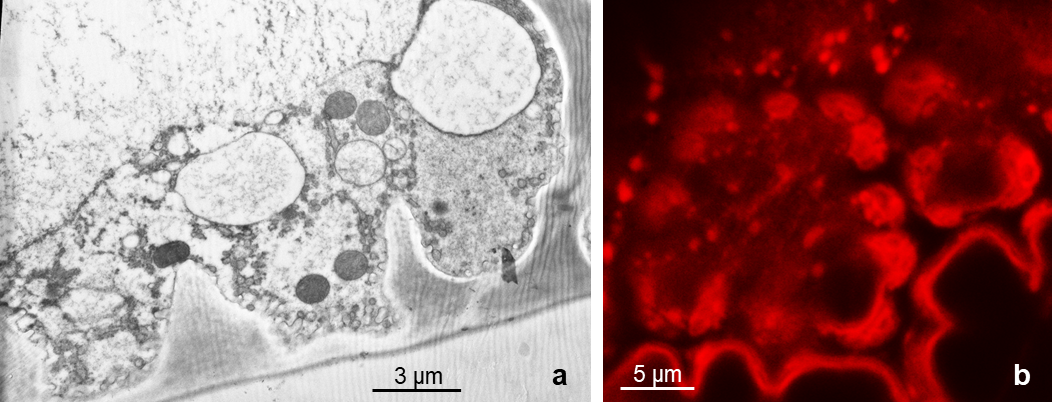

Supplement: Supplementary file 2 — High Resolution (TIF 2695 kb) [file 709_2021_1667_MOESM1_ESM.tif]

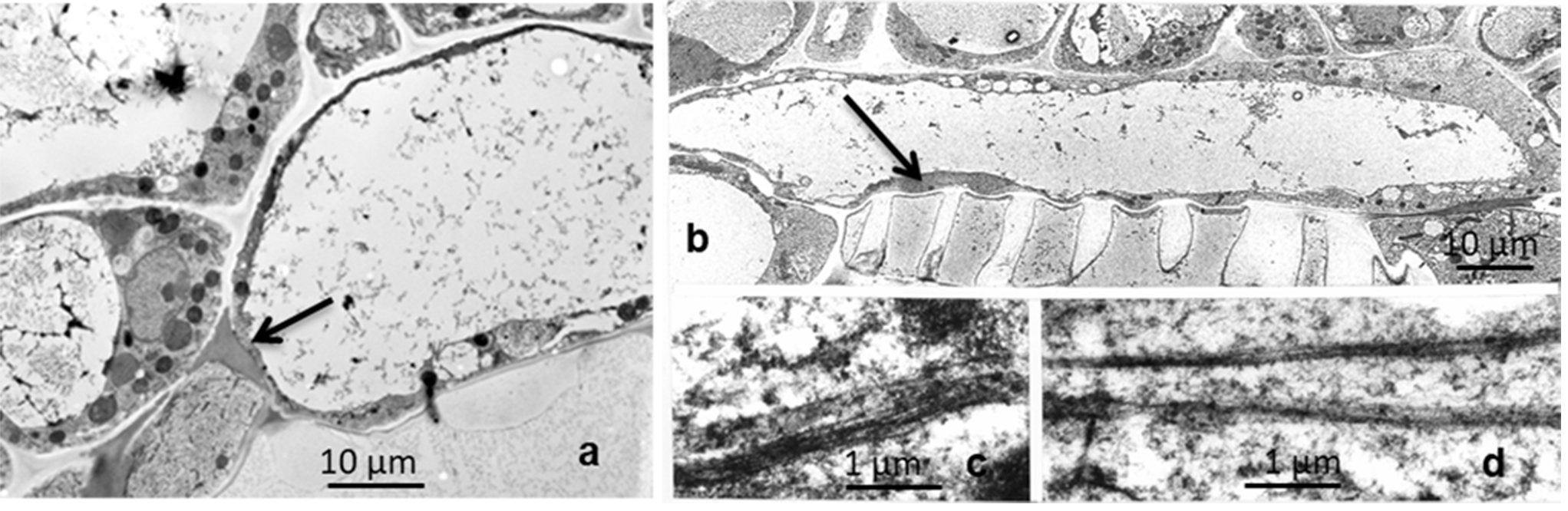

Supplement: Supplementary file 3 — Endodermis of the glandular head. Endodermoid cells are short in the apical zone of the gland head (a) and flat and elongated along the flanks (b). They curve out at the base of the gland head. The radial walls between endodermal cells and the walls bordering the tracheids of the core are impregnated (arrows). The cytoplasm is well preserved despite the deep distance for freezing, and bundles of actin microfilaments account for the high mobility of organelles observed in live cell imaging (c, d) (PNG 1013 kb) [file 709_2021_1667_Fig2_ESM.png]

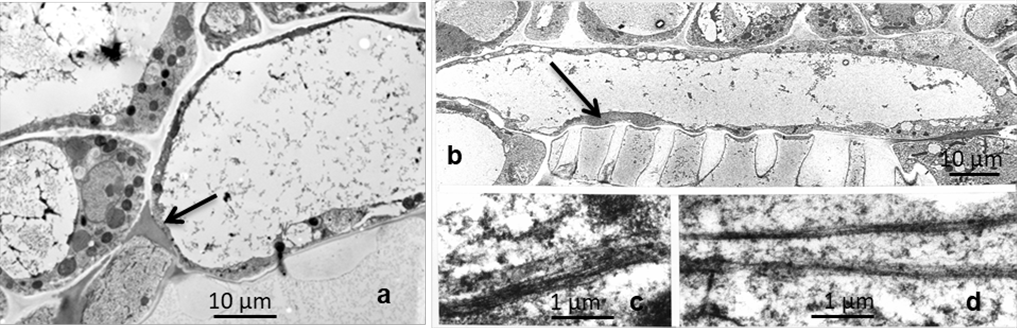

Supplement: Supplementary file 4 — High Resolution (TIF 2327 kb) [file 709_2021_1667_MOESM2_ESM.tif]

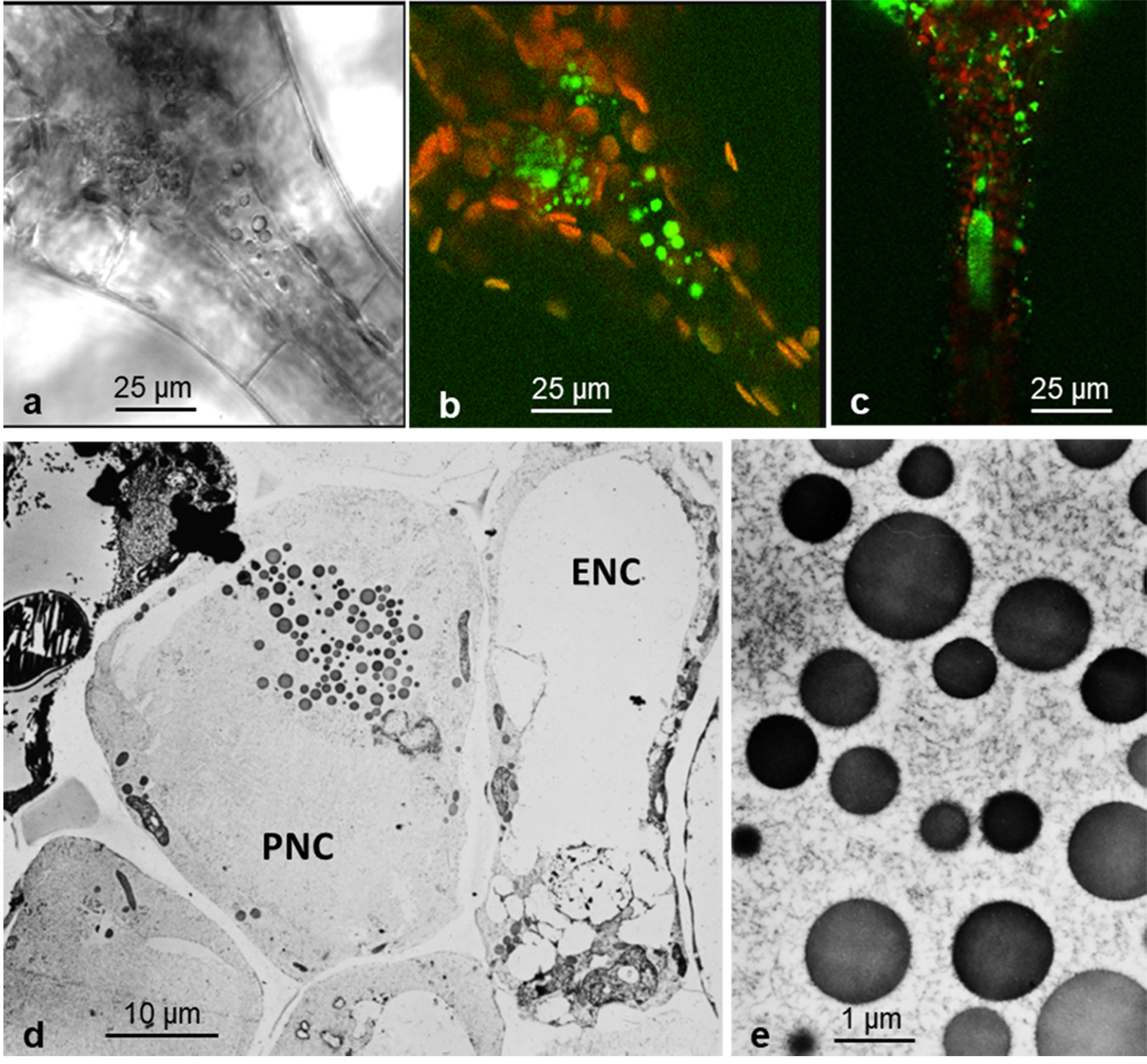

Supplement: Supplementary file 5 — Neck region of Drosera tentacles. Homogenous round bodies cluster in the vacuoles of parenchymal neck cells (PNC) and parenchymal stalk cells (PSC), but not in the vacuoles of epidermal neck cells (ENC) and epidermal stalk cells (ESC). They move in Brownian motion, as seen in bright-field microscopy (a) and emit green fluorescence; chloroplasts shine in red (b). In sections from TEM, they reveal their osmiophilic nature (d, e). Occasionally also the vacuoles of parenchymal stalk cells emit diffuse green fluorescence with a similar spectrum as the globular structures (c) (PNG 2624 kb) [file 709_2021_1667_Fig3_ESM.png]

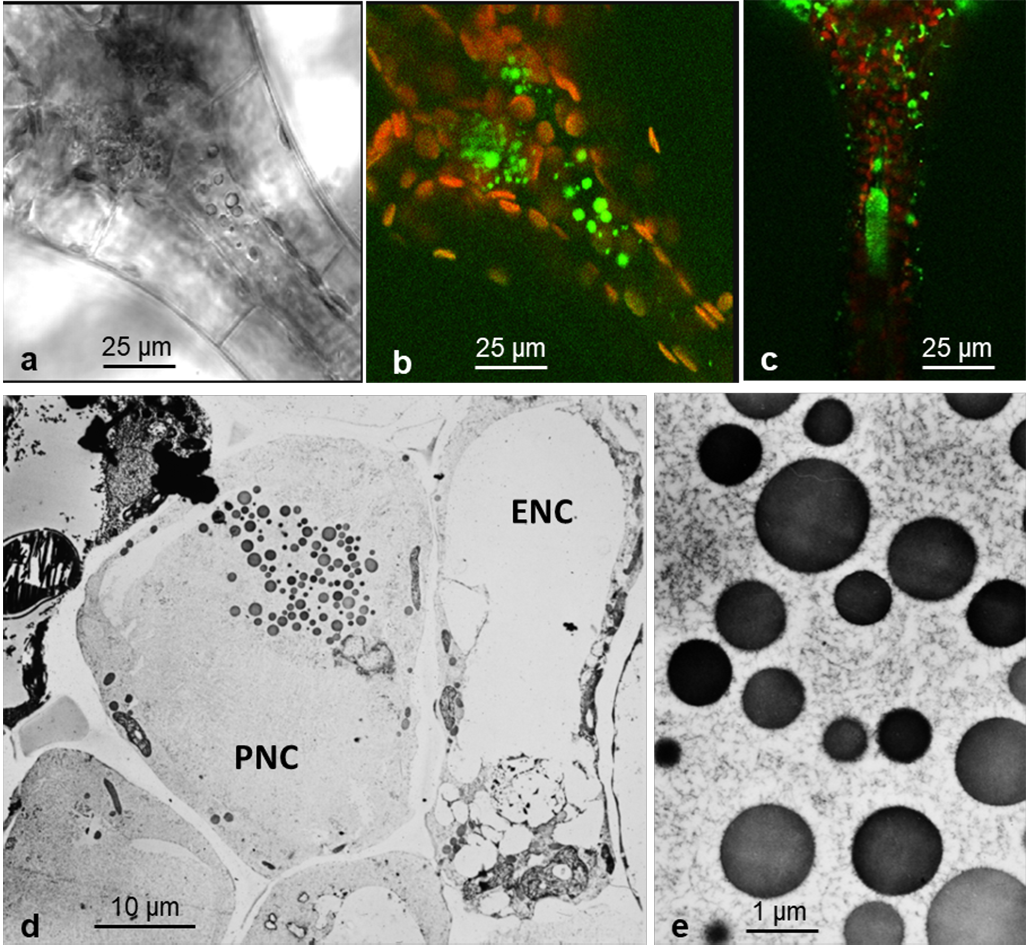

Supplement: Supplementary file 6 — High Resolution (TIF 6519 kb) [file 709_2021_1667_MOESM3_ESM.tif]

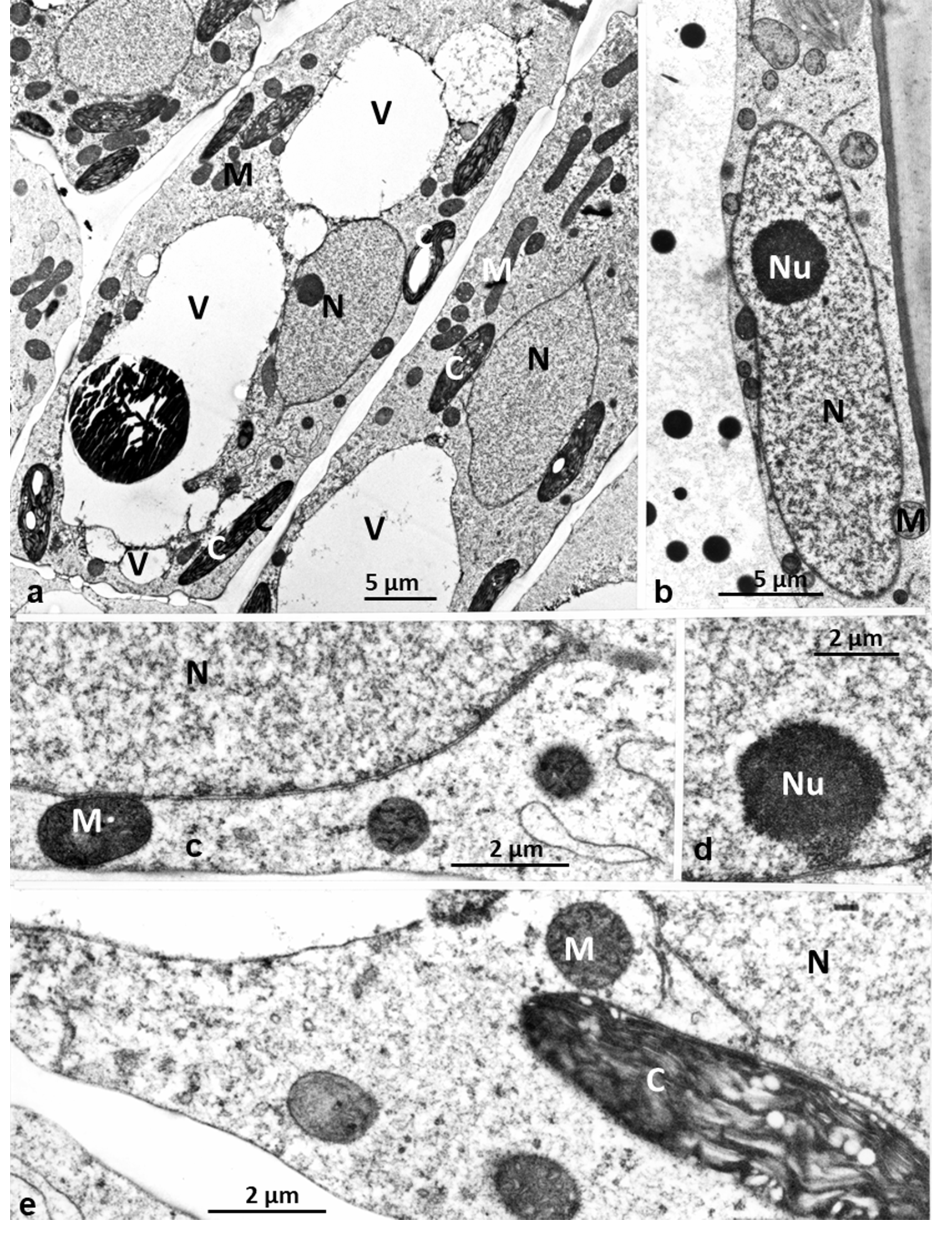

Supplement: Supplementary file 7 — Epidermal cells of the neck and the stalk of Drosera tentacles. Organelle morphology is well preserved after High-Pressure Freezing. These organelles are in close contact with each other. In BSA-treated cells, the large central vacuole is disintegrated into several parts (a). Fixation and substitution cause dark osmiophilic precipitations. Chloroplast (C); Mitochondrion (M); Nucleus (N); Nucleolus (Nu); Cell sap vacuole (V) (PNG 2979 kb) [file 709_2021_1667_Fig4_ESM.png]

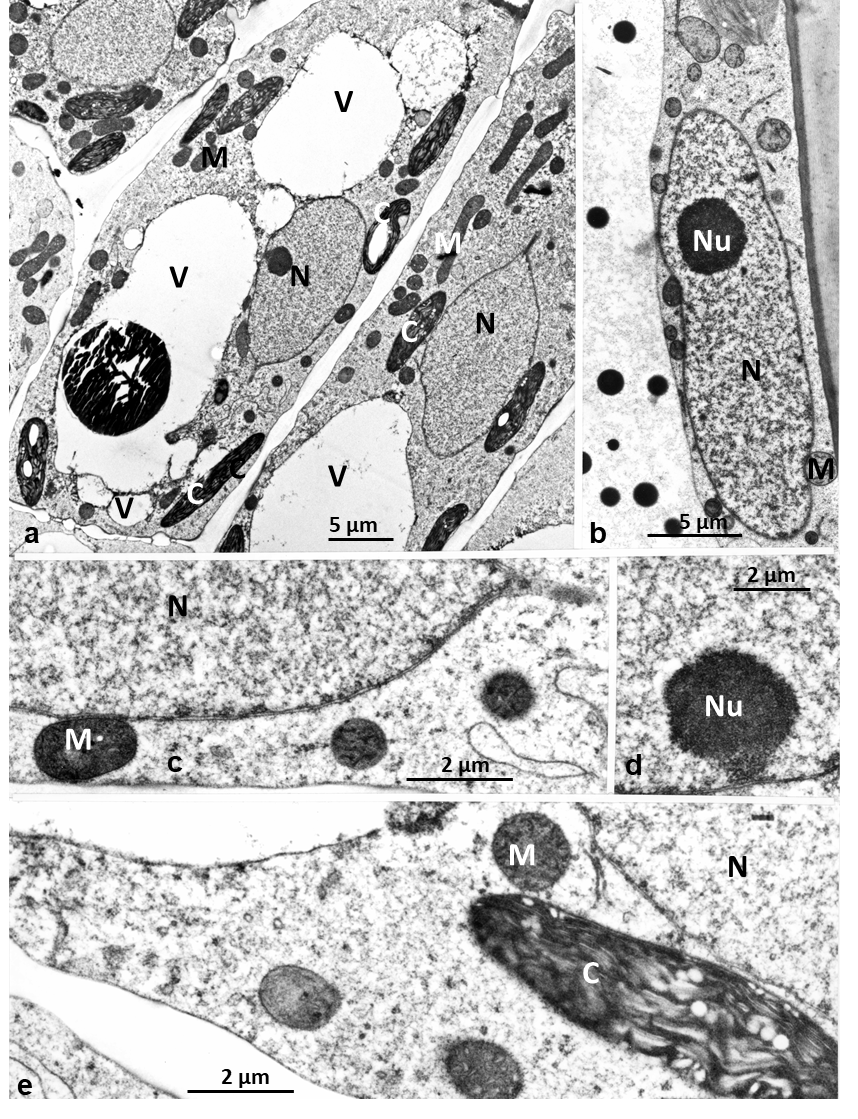

Supplement: Supplementary file 8 — High Resolution (TIF 6343 kb) [file 709_2021_1667_MOESM4_ESM.tif]

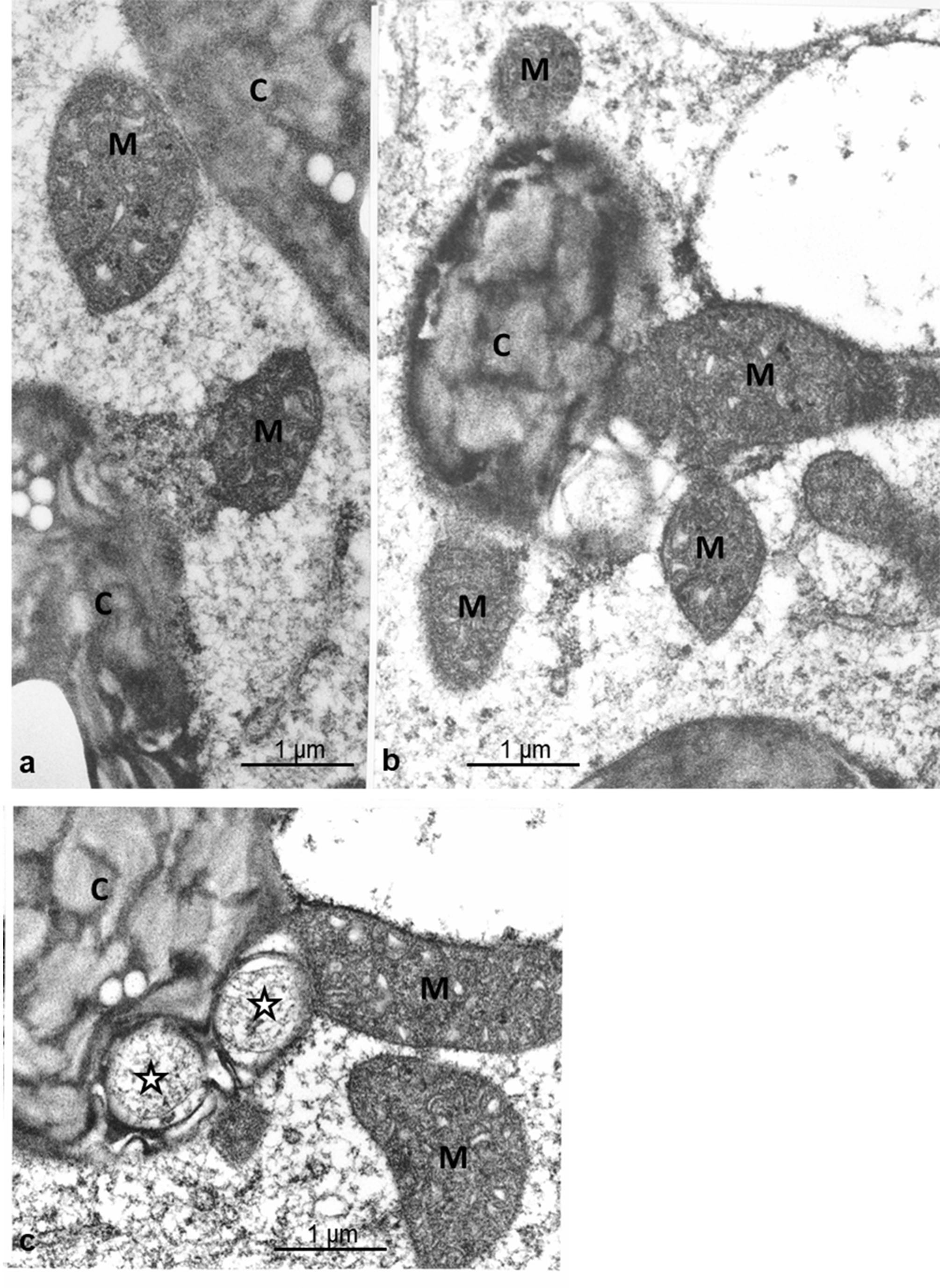

Supplement: Supplementary file 9 — Organelle associations captured by freeze fixation. a, b, c: Chloroplasts (C) are in intimate contact with mitochondria (M); Pleiomorphic chloroplasts engulf portions of cytoplasm (asterisk); (c) (PNG 2941 kb) [file 709_2021_1667_Fig5_ESM.png]

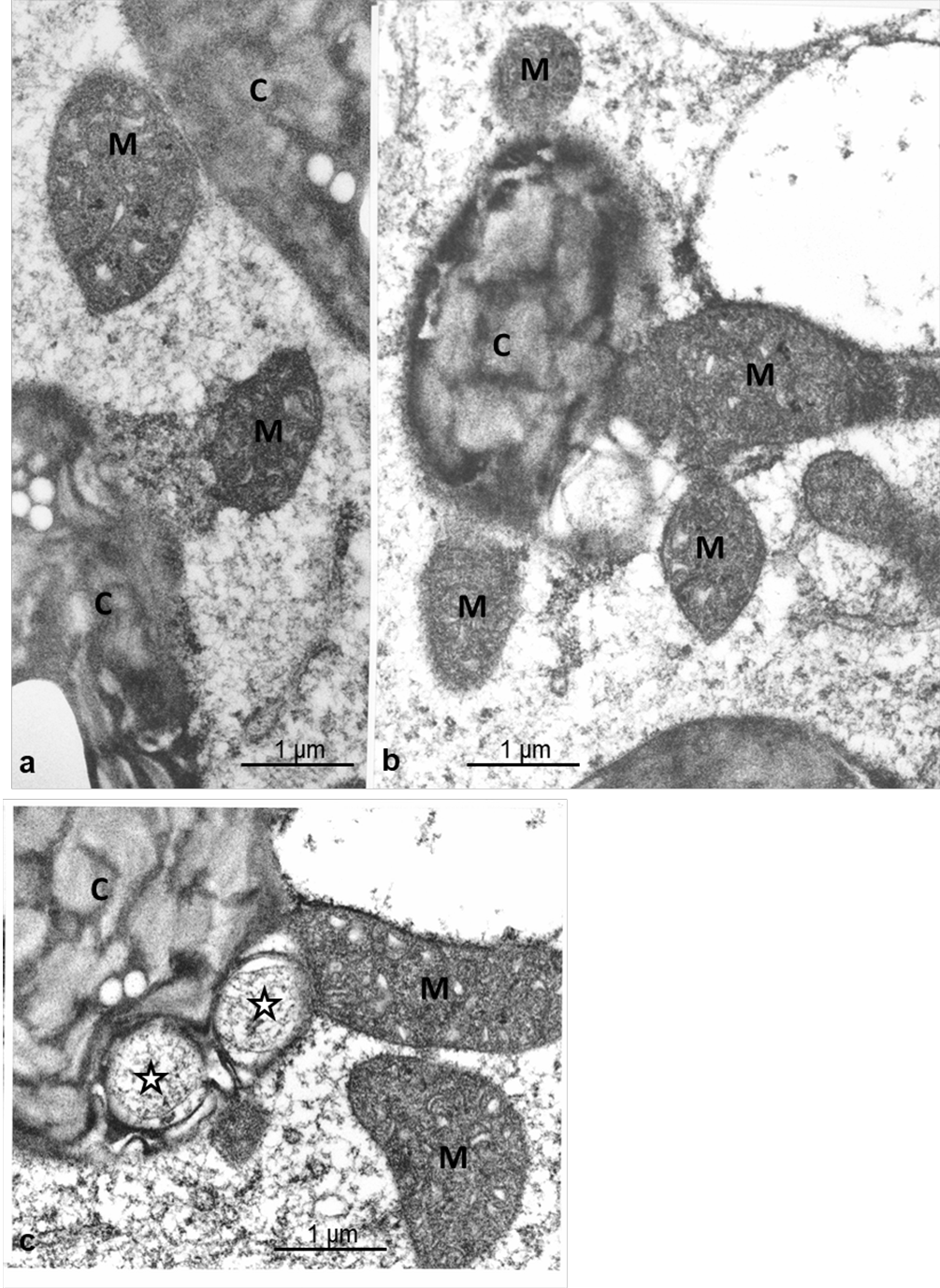

Supplement: Supplementary file 10 — High Resolution (TIF 8783 kb) [file 709_2021_1667_MOESM5_ESM.tif]

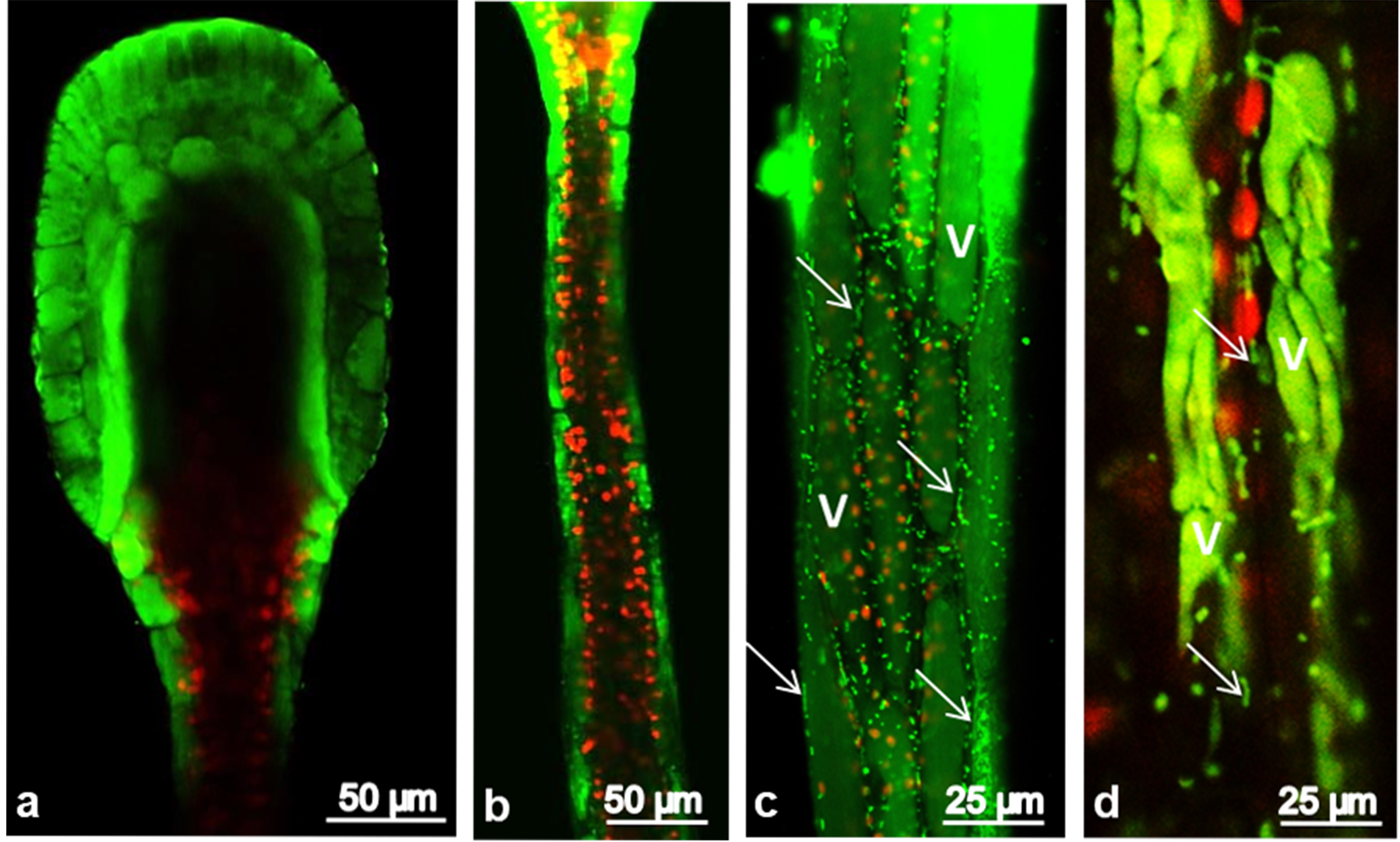

Supplement: Supplementary file 11 — Staining of Drosera tentacles with DiOC6 results in labeling of the cell sap vacuoles, in addition to mitochondria. In the gland head, outer and inner gland cells as well as endodermoid cells are clearly depicted (a). The labeling progresses to the stalk along epidermal stalk cells (b). In the epidermis, round and elongated mitochondria (arrows) possess fluorescence in an otherwise unstained cytoplasm, together with the vacuole (V). In untreated cells, the vacuole fills the cell (c), whereas in BSA-treated cells the vacuole is disintegrated into tubes and vesicles shining brightly within the swollen unstained cytoplasm (d) (PNG 1502 kb) [file 709_2021_1667_Fig6_ESM.png]

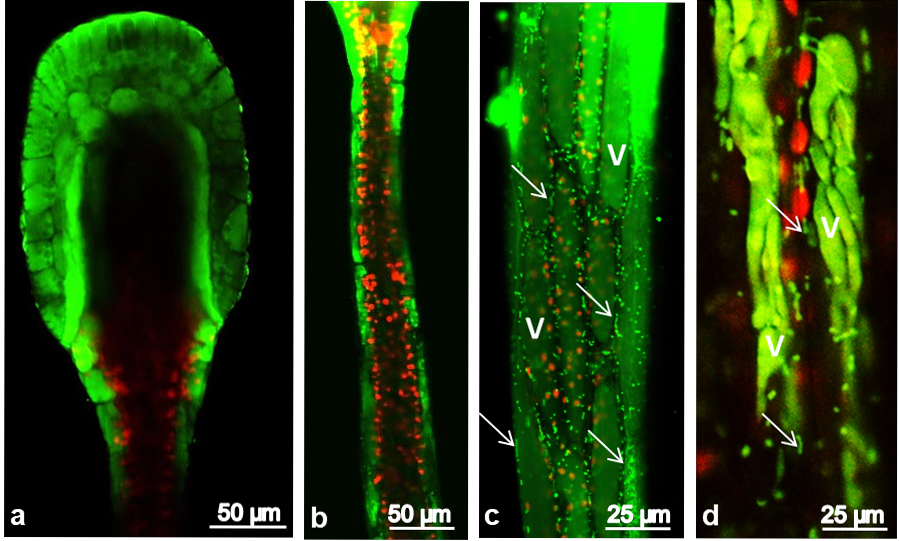

Supplement: Supplementary file 12 — High Resolution (TIF 2923 kb) [file 709_2021_1667_MOESM6_ESM.tif]
